# Topical rhubarb charcoal-crosslinked chitosan/silk fibroin sponge scaffold for the repair of diabetic ulcers improves hepatic lipid deposition in db/db mice via the AMPK signalling pathway

**DOI:** 10.1186/s12944-024-02041-z

**Published:** 2024-02-20

**Authors:** Qi Tan, Qifeng He, Ze Peng, Xin Zeng, Yuzhe Liu, Dong Li, Shang Wang, Jianwei Wang

**Affiliations:** 1 Chongqing Key Laboratory of Traditional Chinese Medicine for Prevention and Cure of Metabolic Diseases, College of Traditional Chinese Medicine, Chongqing Medical University, Chongqing, 400016 China; 2 College of Basic Medical Sciences, Chongqing Medical University, Chongqing, 400016 China; 3 Chongqing College of Traditional Chinese Medicine, Chongqing, 402760 China

**Keywords:** Rhubarb charcoal, Type 2 diabetes, AMPK signalling pathway, Hepatic lipid deposition

## Abstract

**Background:**

Type 2 diabetes mellitus (T2DM) is closely linked to metabolic syndrome, characterised by insulin resistance, hyperglycaemia, abnormal lipid metabolism, and chronic inflammation. Diabetic ulcers (DUs) comprise consequential complications that arise as a result of T2DM. To investigate, db/db mice were used for the disease model. The findings demonstrated that a scaffold made from a combination of rhubarb charcoal-crosslinked chitosan and silk fibroin, designated as RCS/SF, was able to improve the healing process of diabetic wounds in db/db mice. However, previous studies have primarily concentrated on investigating the impacts of the RSC/SF scaffold on wound healing only, while its influence on the entire body has not been fully elucidated.

**Material and methods:**

The silk fibroin/chitosan sponge scaffold containing rhubarb charcoal was fabricated in the present study using a freeze-drying approach. Subsequently, an incision with a diameter of 8 mm was made on the dorsal skin of the mice, and the RCS/SF scaffold was applied directly to the wound for 14 days. Subsequently, the impact of RCS/SF scaffold therapy on hepatic lipid metabolism was assessed through analysis of serum and liver biochemistry, histopathology, quantitative real-time PCR (qRT-PCR), immunohistochemistry, and Western blotting.

**Results:**

The use of the RCS/SF scaffold led to an enhancement in the conditions associated with serum glucolipid metabolism in db/db mice. An assessment of hepatic histopathology further confirmed this enhancement. Additionally, the qRT-PCR analysis revealed that treatment with RCS/SF scaffold resulted in the downregulation of genes associated with fatty acid synthesis, fatty acid uptake, triglyceride (TG) synthesis, gluconeogenesis, and inflammatory factors. Moreover, the beneficial effect of the RCS/SF scaffold on oxidative stress was shown by assessing antioxidant enzymes and lipid peroxidation. Additionally, the network pharmacology analysis verified that the adenosine monophosphate-activated protein kinase (AMPK) signalling pathway had a vital function in mitigating non-alcoholic fatty liver disease (NAFLD) by utilizing *R. officinale.* The measurement of AMPK, sterol regulatory element binding protein 1 (SREBP1), fatty acid synthase (FASN), and acetyl CoA carboxylase (ACC) gene and protein expression provided support for this discovery. Furthermore, the molecular docking investigations revealed a robust affinity between the active components of rhubarb and the downstream targets of AMPK (SREBP1 and FASN).

**Conclusion:**

By regulating the AMPK signalling pathway, the RCS/SF scaffold applied topically effectively mitigated hepatic lipid accumulation, decreased inflammation, and attenuated oxidative stress. The present study, therefore, emphasises the crucial role of the topical RCS/SF scaffold in regulating hepatic lipid metabolism, thereby confirming the concept of "external and internal reshaping".

**Supplementary Information:**

The online version contains supplementary material available at 10.1186/s12944-024-02041-z.

## Introduction

The most prevalent chronic liver condition globally is non-alcoholic fatty liver disease (NAFLD), which impacts approximately a quarter of the adult population [1]. Metabolic disorders like type 2 diabetes mellitus (T2DM) and obesity are closely associated with NAFLD. NAFLD is characterised by hepatic steatosis, insulin resistance, oxidative stress, and chronic inflammation [2, 3]. The pathological progression of NAFLD initiates with the buildup of lipid in the hepatic tissue (hepatic steatosis) and can progress through various stages such as non-alcoholic steatohepatitis (NASH), fibrosis, cirrhosis, and, in severe instances, hepatocellular carcinoma [4]. The established pathogenic hypotheses, known as the "two-hit model" and "multiple parallel hits", emphasise the interconnected nature of liver steatosis with oxidative stress, inflammation, endoplasmic reticulum stress, and intestinal microbial dysfunction, among other factors [5]. However, NAFLD still lacks approved treatments due to the complex nature of its progression and the presence of various associated co-morbidities [6]. Hence, it is imperative to identify effective prophylactic drugs for NAFLD.

Diabetic foot ulcers (DUs), which are a complication of T2DM, have a high incidence (34% of diabetic patients globally) and present a serious risk to human health [7]. Although current approaches such as debridement, infection control, maintaining a moist wound environment, and decompression are used to treat these ulcers, there are still challenges in achieving optimal efficacy and managing high costs [8, 9]. Consequently, patients often experience negative financial consequences. Traditional Chinese medicine (TCM) has accumulated extensive practical knowledge over thousands of years, resulting in a well-established theoretical framework for external treatments. Several studies have illustrated the positive impacts of TCM and the use of compound external applications in the treatment of DUs [10–13]. These therapies can induce angiogenesis, promote cellular proliferation, and inhibit local inflammatory reactions, thus effectively achieving the objective of managing DUs [14].

TCM and its bioactive components have garnered significant attention in recent years due to their versatility and affordability. Owing to these attributes, TCM-based components have been loaded into hydrogels for the treatment of DUs. Numerous studies have demonstrated that hydrogel composites containing diverse TCM ingredients have potent antioxidant properties and effectively enhance the healing process of diabetic wounds [15, 16]. *Rhubarb officinale* Baill refers to a group of perennial plants belonging to the genus *Rheum L.* within the Polygonaceae family. For thousands of years, rhubarb charcoal, derived from the charring process of rhubarb, has been used in China to treat burns and ulcers. In a previous study, our research group combined rhubarb charcoal with a chitosan/silk fibroin sponge scaffold and a hydrogel, using a novel TCM-based approach [17]. The resulting RCS/SF scaffold was demonstrated to be highly effective in facilitating the healing of diabetic wounds.

The concept of "externally curing internal disease" was first introduced by Wu Shangxian, a physician from the Qing Dynasty. He discovered that although therapy is administered to the body's surface, it can have extensive therapeutic effects on the entire system. According to the principles TCM, a key requirement for achieving the "in vitro drug administration and systemic effect" is the drug's ability to penetrate the skin and directly reach internal organs [18, 19]. In this context, previous studies not only validated the effectiveness of the topical RCS/SF scaffold in promoting wound healing in diabetic individuals, but also revealed surprising reductions in serum TG and blood glucose levels in diabetic mice. The liver, a crucial metabolic organ, plays a central role in maintaining the body's metabolic balance and regulating diverse substances [20]. Furthermore, there is a notable connection between NAFLD and T2DM, both of which are linked to disruptions in hepatic glucolipid metabolism [21]. As a result, the concept of "externally curing internal disease" was expanded to encompass "external and internal reshaping", with a specific emphasis on hepatic glucose and lipid metabolism.

The present investigation seeks to analyze the impact of applying the RCS/SF scaffold topically to improve NAFLD in db/db mice. Specifically, it improved inflammation, oxidative stress, and hepatic lipid accumulation in diabetic mice by activating the AMPK signalling pathway. For the initial time, this research uncovers that the RCS/SF scaffold's potential to boost hepatic lipid accumulation in db/db mice, establishing an experimental basis for the concept of "external and internal reshaping" and presenting a novel approach and concept to enhance disorders in hepatic lipid metabolism.

## Materials and methods

### Preparation of RCS/SF scaffold

Carboxymethyl chitosan (EFL-CMCS-200 K, Mw = 100–200 KDa, with 90% deacetylation, and 80% carboxymethyl substitution) and water-soluble silk fibroin (EFL-SF-001, Mw = 6–10 KDa and 99% purity) were acquired from Suzhou Engineering for Life Technology Co. Ltd. in China. The rhubarb charcoal, sourced from the genuine medicinal plant *Rheum officinale* Bail in Chongqing, was produced by carbonization through stir-frying at a temperature of 200 °C. Subsequently, the silk fibroin/chitosan sponge scaffolds were prepared using a freeze-drying reported in the earlier study [17]. In the same way, scaffolds that carry drugs were formed by utilizing a dual physical cross-linking process to obtain the RCS/SF scaffold. This process included the addition of 100 mg of rhubarb charcoal to the CS/SF mixture.

### Animals

Twelve male db/db mice and six male db/m mice, both 8 weeks, were obtained from Jiangsu Huachuang Xinnuo Pharmaceutical Technology Limited Company (Jiangsu, China). In the animal facility of Chongqing Medical University, the mice were accommodated in Specific Pathogen-Free facilities. Each mouse was kept in its own cage at a temperature of 22 ± 2 °C, and a 12-h light/dark cycle was maintained.

### Experimental groups

After a week of adaptation, male db/db mice were randomly assigned to two groups: the model group (db/db, *n* = 6) and the administration group (RC, *n* = 6). The control group consisted of db/m mice (db/m, *n* = 6). Under isoflurane anaesthesia, a puncture biopsy instrument was used to create an 8-mm diameter wound on the back of each mouse with moderate force [22]. Following that, group RC received RCS/SF scaffold, whereas the remaining two groups were administered normal saline solution at the site of wound. The treatment lasted 14 days, with food and water intake recorded every three days. For precise water intake measurement over a three-day span, water bottles were directly weighed, and intake was calculated by comparing weights before and after the period. All animal experiments complied with the ethical guidelines for animal experimentation and were approved by The Ethics Committee of Chongqing Medical University (Approval No. 2022168).

### Serum biochemical analysis

The biochemical assay kits were used in accordance with the instructions given in the kit manual from Nanjing Jiancheng Bioengineering Institute in Nanjing, China. Specific assays were used to measure the serum concentrations of total cholesterol (TC), low-density lipoprotein cholesterol (LDL-C), high-density lipoprotein cholesterol (HDL-C), TG, and glucose. The measurements were taken for TC (A111-1–1), LDL-C (A113-1–1), HDL-C (A112-1–1), TG (A110-1–1), and glucose (A154-1–1). Using a multi-function microplate reader, the TC, TG, and glucose absorbance values were measured at 505 nm, 510 nm, and 505 nm wavelengths, respectively. Furthermore, the measurements of LDL-C and HDL-C were conducted at a wavelength of 546 nm. The utilized kits were exclusively sourced from Nanjing Jiancheng Bioengineering Institute (Nanjing, China).

### Liver biochemical analysis

Nanjing Jiancheng Bioengineering Institute provided the aspartate aminotransferase (AST) (C010-2–1), alanine aminotransferase (ALT) (C009-2–1) and TG (A110-1–1). The test liquid was prepared following the instructions given in the kit with strict adherence. In summary, around 30 mg of liver tissue was measured, and then mixed with nine times the volume of phosphate buffer saline (0.1 mol/L pH 7.4), maintaining a ratio of 1 g to 9 ml. Grinding beads were then added to ensure complete homogenization of the tissues. The mixture was placed on the Four-Dimensional Rotating Mixer (Beyotime, Shanghai, China) and left to incubate overnight at a temperature of 4 °C. Subsequently, the supernatant was collected after homogenizing and centrifuging the liver tissues. To normalize, the protein concentration in liver tissue was determined using a BCA Protein Concentration Assay kit (#P0010, Beyotime, Shanghai, China). Obtaining the absorbance measurements for ALT, AST, and TG involved utilizing a multi-function microplate reader configured to operate at a wavelength of 510 nm.

### Measurement of antioxidant enzymes and lipid peroxidation

Approximately 30 mg of liver tissue homogenate was measured, and the liquid portion was obtained after centrifugation. Afterwards, the hepatic levels of Catalase (CAT), Malondialdehyde (MDA), Total Superoxide Dismutase (SOD) and NAD^+^/NADH were evaluated using CAT, MDA(A007-1–1, A003-1–2, Nanjing, China), SOD, and NAD^+^/NADH assay kits (S0101S, S0175, Beyotime Biotechnology, Shanghai, China) as per the provided instructions.

### Hepatic histopathological analysis

The mouse liver tissue was fixed using a 4 ％ paraformaldehyde solution. Afterwards, it was dried out, encased in paraffin, and cut into sections. Subsequently, these samples were sliced into 4 μm sections using a manual rotary microtome (Leica RM2235, Wetzlar, German) and then stained with haematoxylin and eosin (H&E). The staining procedure yielded a blue colouration in the nucleus and a red colouration in the cytoplasm. Furthermore, the combined occurrence of steatosis, ballooning, and lobular inflammation was evaluated utilizing the NAFLD activity score (NAS) algorithm [23]. Conversely, the liver glycogen content was assessed by employing periodic acid-Schiff (PAS) staining. The paraffin slices were moisturized, then subjected to periodic acid and Schiff's reagent for 5 min and 15 min, respectively. They were then stained with haematoxylin for 2 min. The glycogen exhibited a purplish-red hue, whereas the nucleus was stained with a blue colour. The liver tissue was initially preserved in a 4 $\mathrm{\%}$ paraformaldehyde solution. It was then subjected to a process of gradual removal of water using a sucrose solution ranging from 30 to 10%. Finally, the tissue was embedded using TissueTek OCT Compound. The tissue was subsequently divided into 6 μm slices using a cryotome (Leica CM1860, Wetzlar, German) and then treated with Oil Red O staining. The Oil Red O staining was conducted following the protocol that had been previously established [24]. The Oil Red O working solution was prepared by dissolving 1 g of Oil Red O powder in 100 mL of isopropanol, which was then combined with distilled water in a 3:2 ratio. Following the filtration process, the solution was subjected to staining for a duration of 10 min. The process of differentiation was performed using a solution containing 60% isopropanol, while the nuclei were stained with haematoxylin as a counterstain. The findings revealed the presence of red lipids and blue nuclei. Furthermore, the liver underwent examination for pathological alterations utilizing a light microscope (Olympus BX51, Tokyo, Japan) and the findings were documented at both 200X and 400X magnifications. The liver tissue was additionally examined for the accumulation of glycogen and lipid droplet using the software called National Institutes of Health Image J (NIH, USA). The analysis results were quantified as the proportion of glycogen and lipid droplets that were present in the liver tissue relative to the total area of the visual field.

### Immunohistochemistry

Paraffin slices of liver tissues measuring 4 μm were subjected to a meticulous process, which involved de-paraffinisation, hydration, and a 10-min treatment with endogenous peroxidase blockers. Subsequently, the slices were subjected to antigen repair for 5 min. After a 30-min interval with bovine serum albumin (BSA), the slices were incubated overnight at 4 °C with rabbit anti-TNFα antibody (GB11188-100, Servicebio, Wuhan, China, 1:300). Afterward, the liver sections were exposed to secondary antibodies that were linked with horseradish peroxidase for a period of 30 min at room temperature. Following this, the sections were stained using a DAB working solution (AFIHC004, AiFang biological, Changsha, China) and subsequently counterstained with haematoxylin. Furthermore, the sections were analyzed utilizing a light microscope (Olympus BX51, Tokyo, Japan) and pictures were captured at both 100X and 400X magnification. Afterwards, the images were examined and quantitatively utilizing the ImageJ software (NIH, USA).

### Quantitative real-time PCR

Total RNA extraction from the liver was performed using AG RNAex Pro Reagent (AG21102, Accurate Biotechnology, Changsha, China). The RNA concentration was determined using the NanoDrop 2000 spectrophotometer and adjusted to a standard level for all samples. Afterwards, complementary deoxyribonucleic acid (cDNA) was produced using the Evo M-MLV reverse transcription Premix kit (AG11705, Accurate Biotechnology, Changsha, China) for quantitative real-time PCR. Quantitative RT-PCR reactions were prepared using the premixed qPCR kit (AG11740, Accurate Biotechnology, Changsha, China) containing SYBR Green Pro Taq HS. The CFX-96 real-time PCR system (BioRad, Hercules, CA, USA) was used to amplify these reactions. Quantification of mRNA levels was performed using the 2^−ΔΔCt^ method and normalised to the ribosomal protein lateral stalk subunit P0 (*Rplp0*), which served as a control gene. The primer sequences utilised in the experiment are presented in Table S1.

### Western blotting

Liver samples weighing 30 mg were homogenized using RIPA buffer (Sangon Biotech, Shanghai, China) supplemented with protease and phosphatase inhibitors. The protein samples were homogenised for 3 min using a TissueLyser. Subsequently, they were centrifuged at a 12,000 rpm and a temperature of 4℃ for 10 min (#5810, Eppendorf, Hamburg, Germany). After collecting the resulting supernatants, the BCA protein concentration determination kit (#P0010, Beyotime, Shanghai, China) was used to quantify the total protein. Subsequently, the samples were subjected to separation using 10–12% SDS-PAGE and TGX Stain-free gels (1,610,183, BioRad, Hercules, CA, USA), and subsequently transferred to PVDF membranes (Millipore, Bedford, MA, USA). For a duration of 1 h, the membranes were blocked in a solution of Tris Buffered Saline with Tween (TBST), containing 5% non-fat dry milk (or 5% BSA for the phosphor-AMPK). Afterwards, the samples were left to incubate overnight at a temperature of 4 °C with a primary antibody. The blot was then subjected to treatment with horseradish peroxidase-conjugated anti-IgG. Detection was carried out using ECL (BL520A, Biocharp, Beijing, China). Moreover, the stain-free technology (BioRad) was employed for normalizing total protein concentration [25]. Image acquisition was performed using the ChemiDoc Imaging System (BioRad, Hercules, CA, USA), and the ImageJ software (NIH, USA) was utilized for quantitative analysis of the target bands' intensities. The antibodies utilized in this investigation were AMPKα (AF6423, Affinity, Changzhou, China, 1:1000), Phospho-AMPKα^Thr172^ (#AF3423, Affinity, USA, 1:1000), SREBP1 (sc-13551, Santa Cruz, CA, USA, 1:500), FASN (10,624–2-AP, Proteintech, Wuhan, China, 1:5000), and ACC (3676 s, Cell Signaling Technology, Danvers, MA, USA, 1:1000).

### Network pharmacology analysis

To explore the various components, crucial targets, and possible mechanisms of rhubarb in fighting NAFLD, the network pharmacology method was employed. "Rhubarb" and "non-alcoholic fatty liver disease" served as search keywords in relevant databases. Active ingredients of rhubarb were obtained from the Traditional Chinese Medicine Database and Analysis Platform (TCMSP, https://tcmsp-e.com/) database, using a threshold of drug-likeness (DL) ≥ 0.18 as a criterion. The Uniprot database (https://www.uniprot.org/) was utilized to screen these substances and their possible targets. Simultaneously, NAFLD targets were obtained through searches conducted in the OMIM (https://www.omim.org/) and GeneCards (https://www.genecards.org/) databases. Subsequently, by creating a Venn diagram, the shared targets between rhubarb and NAFLD were identified. Furthermore, the core targets were obtained from the STRING (https://string-db.org/) database. Following analyses involved the use of Gene ontology (GO) enrichment and Kyoto Encyclopaedia of Genes and Genome (KEGG) pathway assessments to assess the potential targets.

### Molecular docking analysis

To validate the interaction activities between the active components and targets, AutoDock Vina (1.1.2), a software for molecular docking, was employed. The compounds in mol2 format were obtained from the official TCMSP website, whereas the primary target proteins were retrieved from the PDB database (http://www.rcsb.org/). Proteins were imported into AutoDocktools (v1.5.6) and saved as "pdbqt". The interaction patterns were then analysed using PyMOL 2.3.0.

### Statistics analysis

GraphPad Prism 9.0 software (GraphPad Software Inc.) was utilized for the statistical analysis. The means ± standard errors of the mean (SEM) were used to display all graphical data. Group comparisons were analysed using a one-way ANOVA followed by Dunnett's test. Accordingly, the values with a significance level of *P* < 0.05 were statistically significant.

## Results

### RCS/SF scaffold decreases liver weight and liver index in db/db mice

**Fig. 1:**
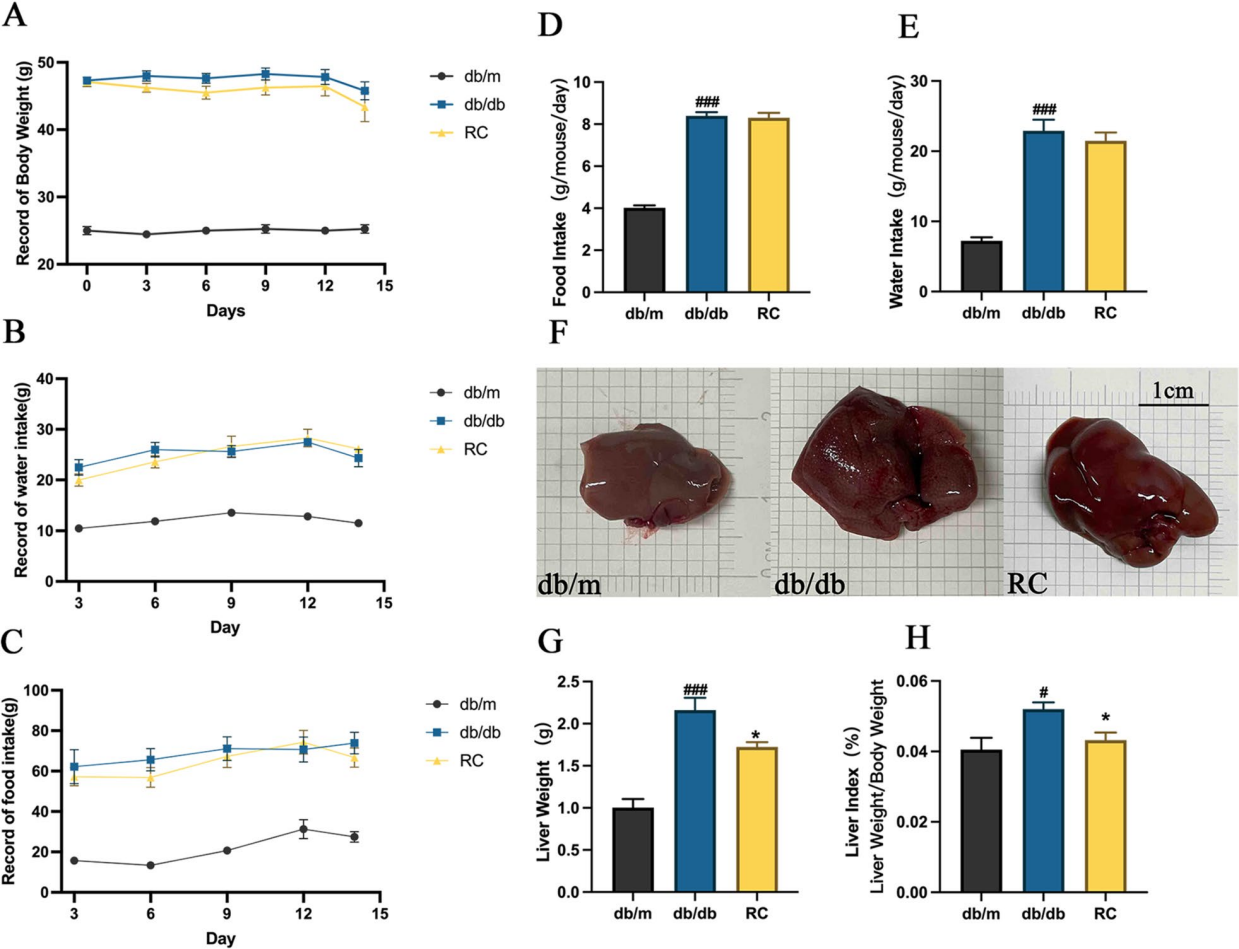
General condition of mice during RCS/SF scaffold treatment. **A** Record of body weight. **B** Record of food intake. **C** Record of water intake. **D** Average daily food intake per mouse. **E** Average daily water intake per mouse. **F** Liver morphology. **G** Liver weight. **H** Liver index. The values reported in the figure represent the means **± **SEM (*n* = 6). ###*P* < 0.001, #*P* < 0.05 compared with the db/m group; *P < 0.05 compared with the RC group

The diabetic mice displayed noticeably increased body weight, food intake, and water intake compared to the db/m mice over a span of 14 days (Figs. 1A-C). However, the administration of RCS/SF scaffold did not have any noticeable effect on the overall condition of the db/db mice (Figs. 1A-C). In addition, the graphs illustrating the average consumption of food and water showed no discernible difference between the db/db group and the RC group (Figs. 1D, E), suggesting that diabetic mice had similar energy intake. Moreover, the hepatic morphological photographs clearly demonstrated that the db/m group displayed a sleek and supple texture. On the other hand, the db/db group exhibited a significantly larger size, characterised by a surface adorned with conspicuous white, shiny, granular protrusions. The application of RCS/SF scaffold treatment effectively reduced the prominent roughness on the liver surface of diabetic mice, while also reducing the enlarged size of the liver (Fig. 1F). Moreover, the credibility of this result was verified by the significant reduction in liver weight and liver index after the intervention in the RC group. (Figs. 1G, H). The combined results indicate that the use of RCS/SF scaffold in treatment led to a reduction in both liver weight and liver index, without impacting the overall body weight.

### RCS/SF scaffold improves disorders of glucolipid metabolism in db/db mice

**Fig. 2:**
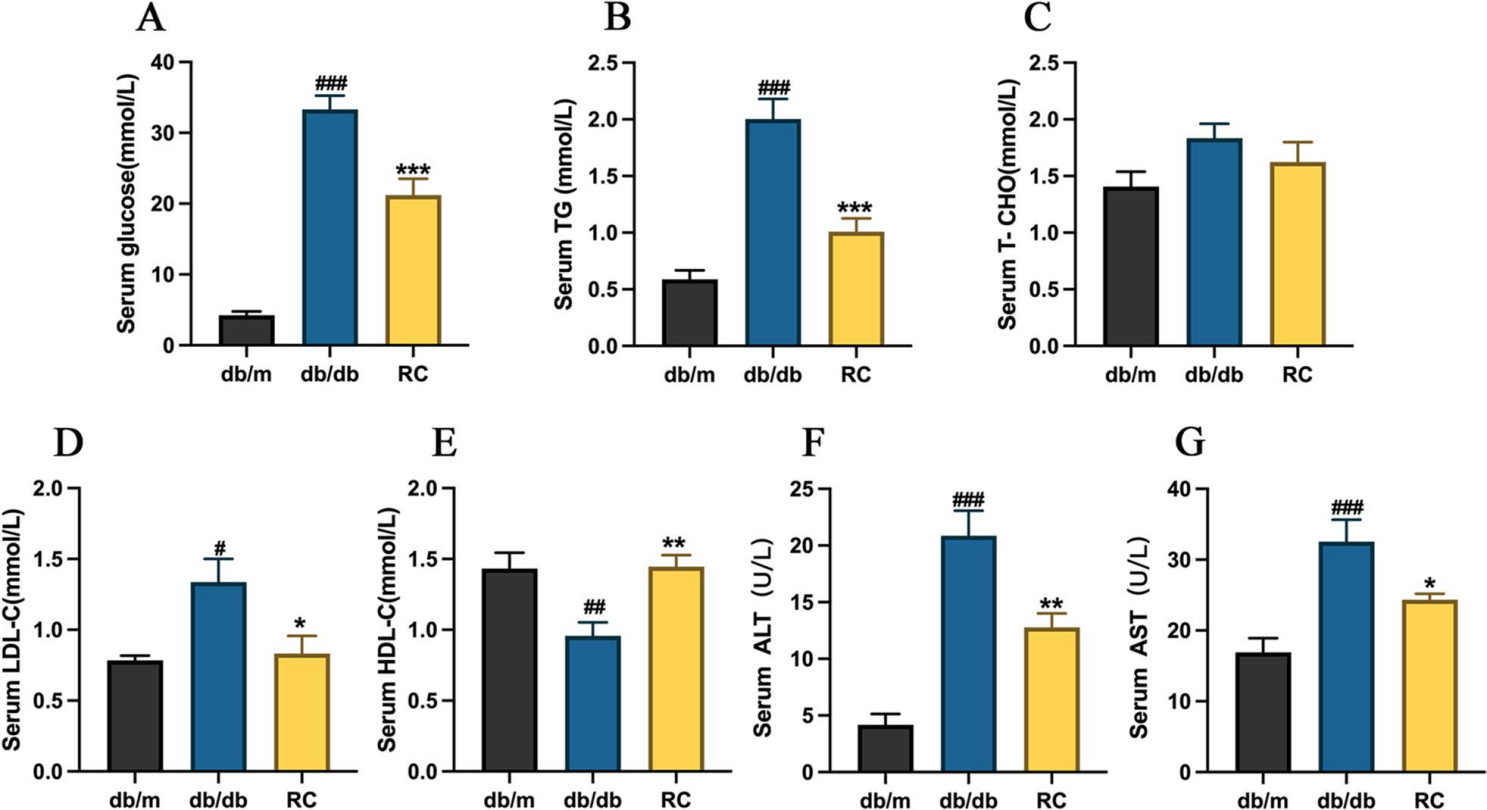
Effect of RCS/SF scaffold treatment on serum biochemical indexes in db/db mice. **A** Serum glucose. **B** Serum triglyceride (TG). **C** Serum total cholesterol (TC). **D** Serum low-density lipoprotein cholesterol (LDL-C). **E** Serum high-density lipoprotein cholesterol (HDL-C). **F** Serum alanine aminotransferase (ALT). **G** Serum aspartate aminotransferase (AST). The values reported in the figure represent the means **± **SEM (*n* = 6). ###*P* < 0.001, ##*P* < 0.01, #*P* < 0.05 compared with the db/m group; ***P < 0.001, **P < 0.01, *P < 0.05 compared with the RC group

The RCS/SF scaffold demonstrated a significant hypoglycaemic effect on diabetic mice. As depicted in Fig. 2A, the serum glucose levels in the RC group were significantly lower compared to the db/db group. No significant difference in serum TC was observed between the RC group and the db/db group in terms of its impact on blood lipids (Fig. 2C). Nevertheless, the levels of serum TG and serum LDL-C were notably reduced in the RC group (Figs. 2B, D). Furthermore, the RCS/SF scaffold treatment successfully restored the decreased HDL-C content in the db/db group (Fig. 2E). Importantly, a notable decrease in serum ALT and AST levels was observed in diabetic mice receiving treatment with RCS/SF scaffold (Figs. 2F, G). To summarise, these findings indicate that the RCS/SF scaffold effectively mitigates glycolipid abnormalities in diabetic mice.

### RCS/SF scaffold ameliorates liver injury and lipid deposition in db/db mice

**Fig. 3:**
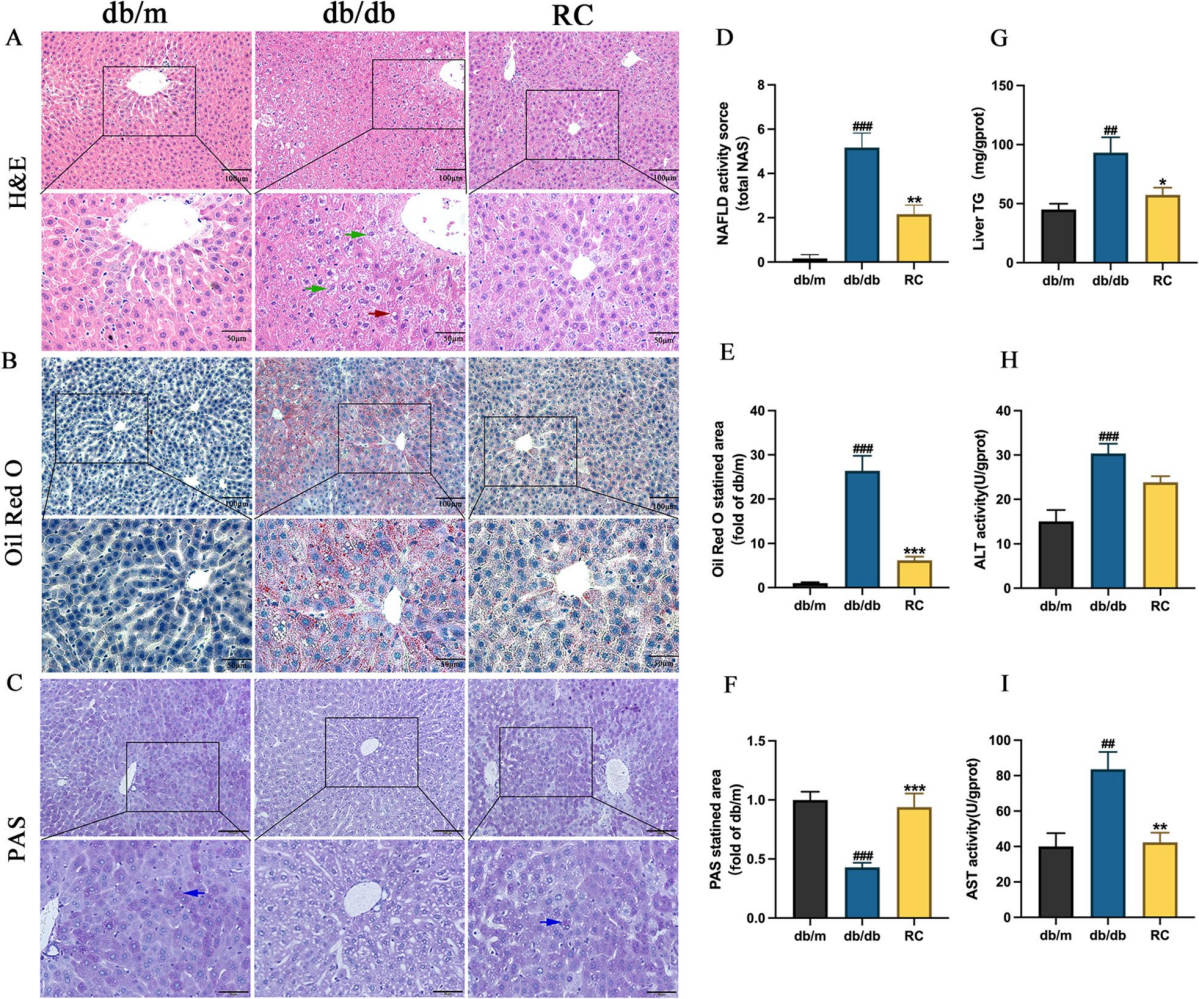
Effects of RCS/SF scaffold on amelioration of liver injury and lipid deposition in db/db mice. **A** H&E staining of liver sections (up row, x 200; down row, x 400). Green arrows indicate ballooning changes, red arrows indicate lipid droplets. **B** Oil-Red O staining of liver sections (up row, x 200; down row, x 400). **C** PAS staining of liver sections (up row, x 200; down row, x 400). Blue arrows indicate glycogen granules. **D** NAFLD activity score (total NASH). **E** Relative quantification of the Oil-Red O staining area. **F** Relative quantification of the PAS staining area. **G** Hepatic TG levels. **H** ALT activity level of liver. **I** AST activity level of liver. The values reported in the figure represent the means **±** SEM (*n* = 6). ####*P* < 0.0001, ###*P* < 0.001, ##*P* < 0.01 compared with the db/m group; ***P < 0.001, **P < 0.01, *P < 0.05 compared with the RC group

To investigate the impact of the RCS/SF scaffold on reducing liver damage and lipid accumulation in db/db mice, a histopathological analysis of the liver was performed. NAFLD encompasses a range of conditions that involve the buildup of fat in the liver (steatosis), inflammation causing cell death (necrotizing inflammation), and mild inflammation resulting in hepatocellular injury [3]. The liver of db/db mice exhibited significant steatosis and ballooning, as evidenced by H&E staining. However, the administration of RCS/SF scaffold mitigated this pathological condition (Figs. 3A, D). The db/db group showed a significant increase and roundness of liver cells, along with a considerable increase in fat droplets, as indicated by the staining with oil red O. Nonetheless, the implementation of RCS/SF scaffold led to a significant decrease in both the dimensions and number of lipid droplets (Figs. 3B, E). Additionally, the PAS staining revealed abundant purplish-red glycogen particles in the liver tissue of db/m mice, whereas a substantial decrease was observed in db/db mice. However, the utilization of the RCS/SF scaffold successfully reinstated the diminished glycogen accumulation (Figs. 3C, F). Meanwhile, the liver biochemistry examination showed a significant reduction in hepatic TG levels in db/db mice treated with the RCS/SF scaffold (Fig. 3G). Furthermore, the liver of db/db mice displayed markedly elevated levels of ALT and AST, which are widely recognized as markers of liver damage, in comparison to db/m mice. Nevertheless, the administration of RCS/SF scaffold treatment effectively inhibited the increase in ALT and AST level (Figs. 3H, I). Overall, these data suggest that the RCS/SF scaffold ameliorates hepatic lipid deposition and reduces liver damage in db/db mice.

### RCS/SF scaffold mitigates hepatic oxidative stress in db/db mice

**Fig. 4:**
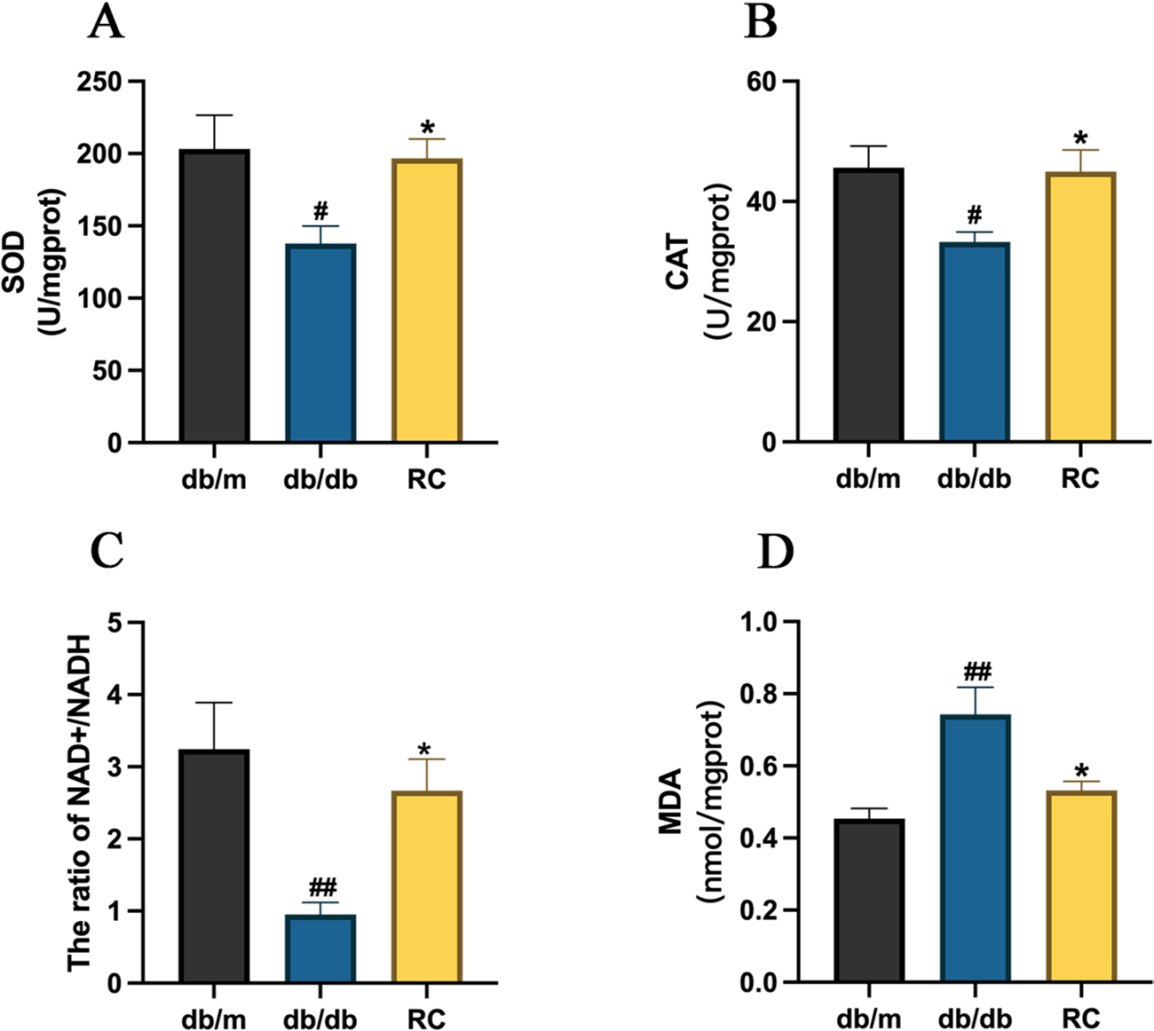
Effect of RCS/SF scaffold on ameliorating hepatic oxidative stress in db/db mice. **A** Hepatic SOD activity. **B** Hepatic CAT activity. **C** Hepatic NAD^+^/NADH activity. **D** Hepatic MDA contents. The values reported in the figure represent the means ± SEM (*n* = 5–6). ##*P* < 0.01, #*P* < 0.05 compared with the db/m group; *P < 0.05 compared with the RC group

A study was conducted to assess the influence of the RCS/SF scaffold on oxidative stress in the livers of db/db mice,, considering the notable contribution of oxidative stress to the progression of NAFLD [26]. As depicted in Fig. 4, in comparison to db/m mice, db/db mice showed a notable reduction in the levels of SOD, CAT, and NAD^+^/NADH in the liver. However, the administration of RCS/SF scaffold resulted in elevated levels of these antioxidant enzymes in the liver tissue (Figs. 4A-C). Furthermore, the findings also indicated a notable decrease in heightened MDA levels in db/db mice after being treated with the RCS/SF scaffold (Fig. 4D). Therefore, the previously mentioned information indicates that the RCS/SF scaffold exerts a safeguarding influence on NAFLD through the regulation of the liver's antioxidant capacity in db/db mice.

### RCS/SF scaffold modulates expression of genes related to glycolipid metabolism in db/db mice

**Fig. 5:**
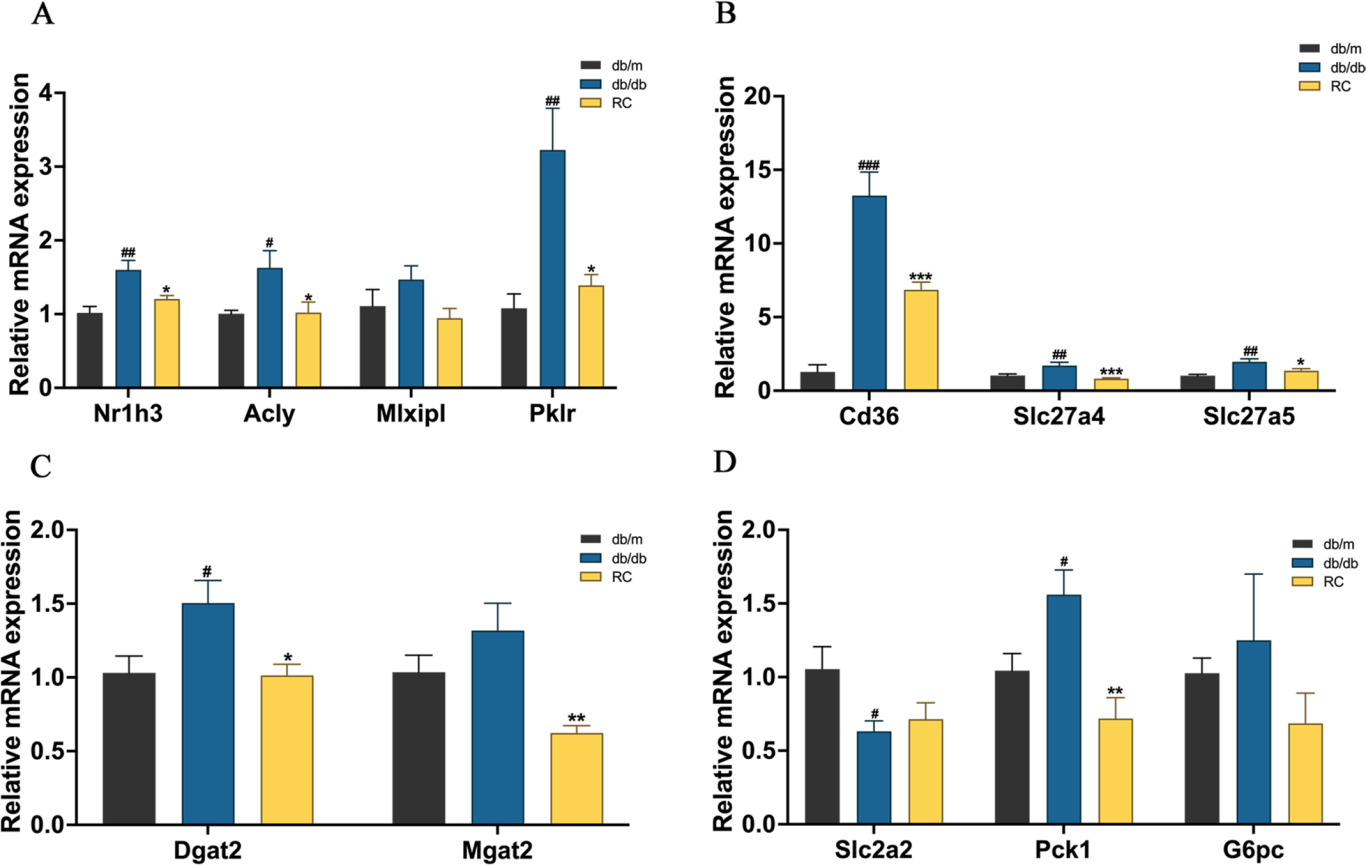
Effect of RCS/SF scaffold on the glycolipid metabolism related gene expression in db/db mice. **A** Relative mRNA expression of the genes involved in fatty acid synthesis. **B** Relative mRNA expression of the genes involved in fatty acid transport. **C** Relative mRNA expression of the genes involved in TG synthesis. **D** Relative mRNA expression of the genes involved glucose metabolism. The values reported in the figure represent the means ± SEM (*n* = 6). ###*P* < 0.001, ##*P* < 0.01, #*P* < 0.05 compared with the db/m group; ***P < 0.001, **P < 0.01, *P < 0.05 compared with the RC group

To examine the impact of the RCS/SF scaffold on decreasing hepatic lipid accumulation in db/db mice, gene expression levels were measured using qRT-PCR for investigation purposes. The results suggest a significant reduction in the mRNA expression of nuclear receptor subfamily 1 group h member 3 (*Nr1h3*), ATP citrate lyase (*Acly*) and pyruvate kinase liver and red blood cell (Lpk, *Pklr*) genes regulated by MLX interacting protein-like (ChRebp, *Mlxipl*) in db/db mice treated with RCS/SF scaffold (Fig. 5A). Furthermore, the RCS/SF scaffold therapy effectively decreased the elevated mRNA levels of genes in db/db mice linked to the transportation of fatty acids, particularly the CD36 molecule (*Cd36*), solute carrier family 27 member 4 (Fatp4, *Slc27a*4), and solute carrier family 27 member 5 (Fatp5, *Slc27a5*) (Fig. 5B). The expression of genes implicated in glucose metabolism was also assessed. Despite not inducing a reversal in the expression of solute carrier family 2-member 2 (Glut2, *Slc2a2*) in db/db mice, the RCS/SF scaffold notably decreased the mRNA expression of phosphoenolpyruvate carboxykinase 1 (Pepck, Pck1) (Fig. 5C). In addition, the levels of diacylglycerol O-acyltransferase 2 (Dgat2) and mannoside acetylglucosaminyltransferase 2 (Mgat2), essential enzymes in TG synthesis, were examined. A notable decrease in the expression of these enzymes was observed when utilizing the RCS/SF scaffold. Taken together, the RCS/SF scaffold influences the activity of genes related to glycolipid metabolism in db/db mice, leading to the inhibition of fatty acid synthesis, fatty acid transport, TG synthesis, and suppression of gluconeogenesis.

### RCS/SF scaffold ameliorates hepatic inflammation in db/db mice

**Fig. 6:**
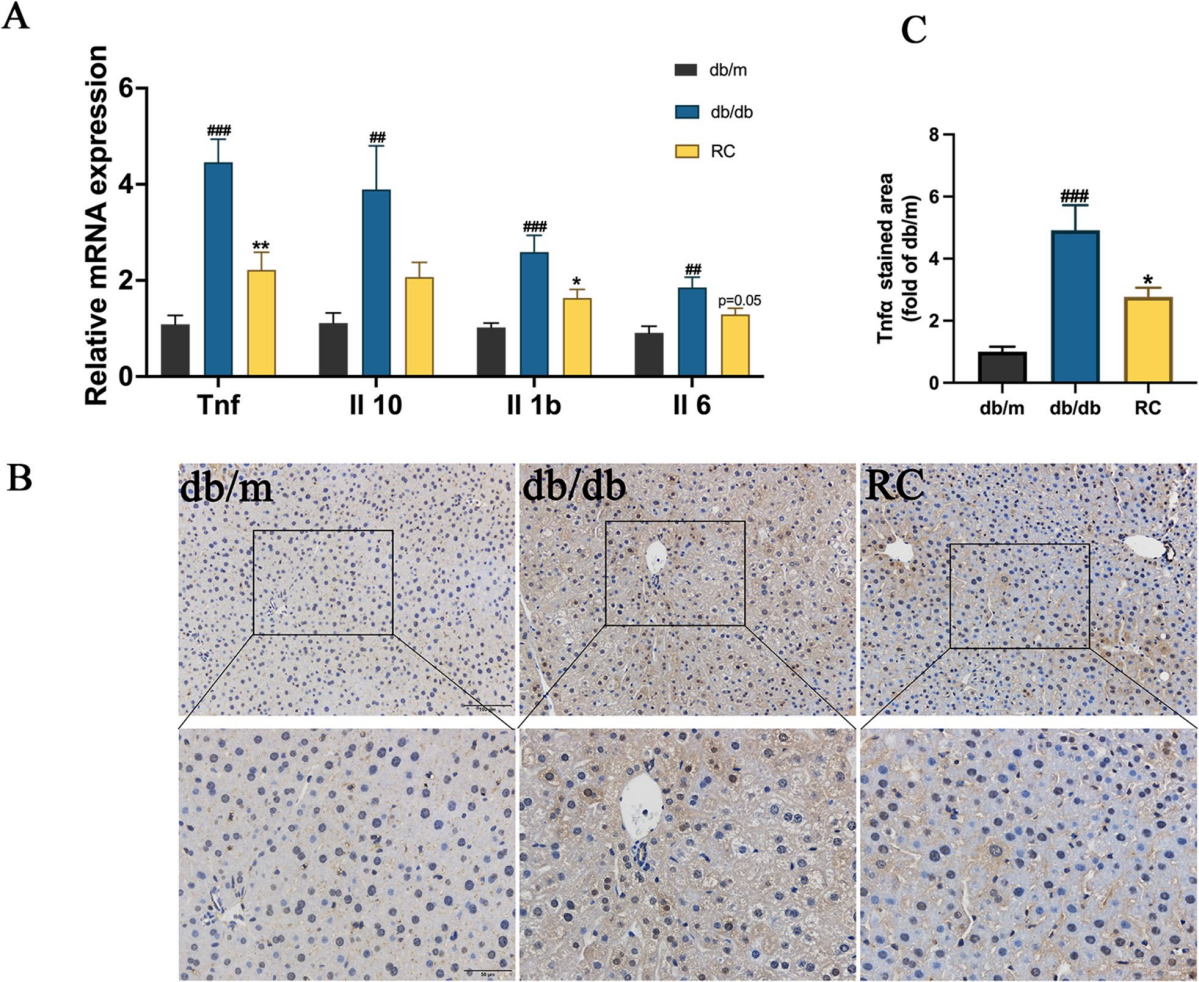
Effect of RCS/SF scaffold on ameliorative hepatic inflammation in db/db mice. **A** Relative mRNA expression of the inflammatory factors (*Tnfα*, *Il-6*, *Il-10*, and *Il-1β*). **B** Immunohistochemical staining of Tnfα (up row, x 200; down row, x 400). **C** Quantitative statistics of Tnfα staining. The values reported in the figure represent the mean ± SEM (*n* = 6). ###*P* < 0.001, ##*P* < 0.01 compared with the db/m group; **P < 0.01, *P < 0.05 compared with the RC group

Given the notable influence of the RCS/SF scaffold on enhancing hepatic function, an examination was carried out to examine its effect on hepatic inflammation. In db/db mice, the qRT-PCR results indicated a notable rise in the mRNA relative expression of inflammatory factors, including tumour necrosis factor-alpha (*Tnfα*), interleukin 6 *(Il-6*), interleukin 10 (*Il-10*), and interleukin 1 beta (*Il-1β*). However, the application of RCS/SF scaffold led to a notable decrease in the levels of *Tnfα* and *Il-1β* expression (Fig. 6A). Furthermore, examination of liver tissue using immunohistochemical analysis demonstrated a notable increase in Tnfα protein expression in the db/db group when compared to the db/m group. Following intervention with the RCS/SF scaffold, its expression exhibited a substantial decrease in comparison to the db/db group (Fig. 6B). As observed, the findings suggest that the RCS/SF scaffold has a positive impact in improving hepatic inflammation.

### RCS/SF scaffold activates AMPK/SREBP1 pathway in db/db mice

**Fig. 7:**
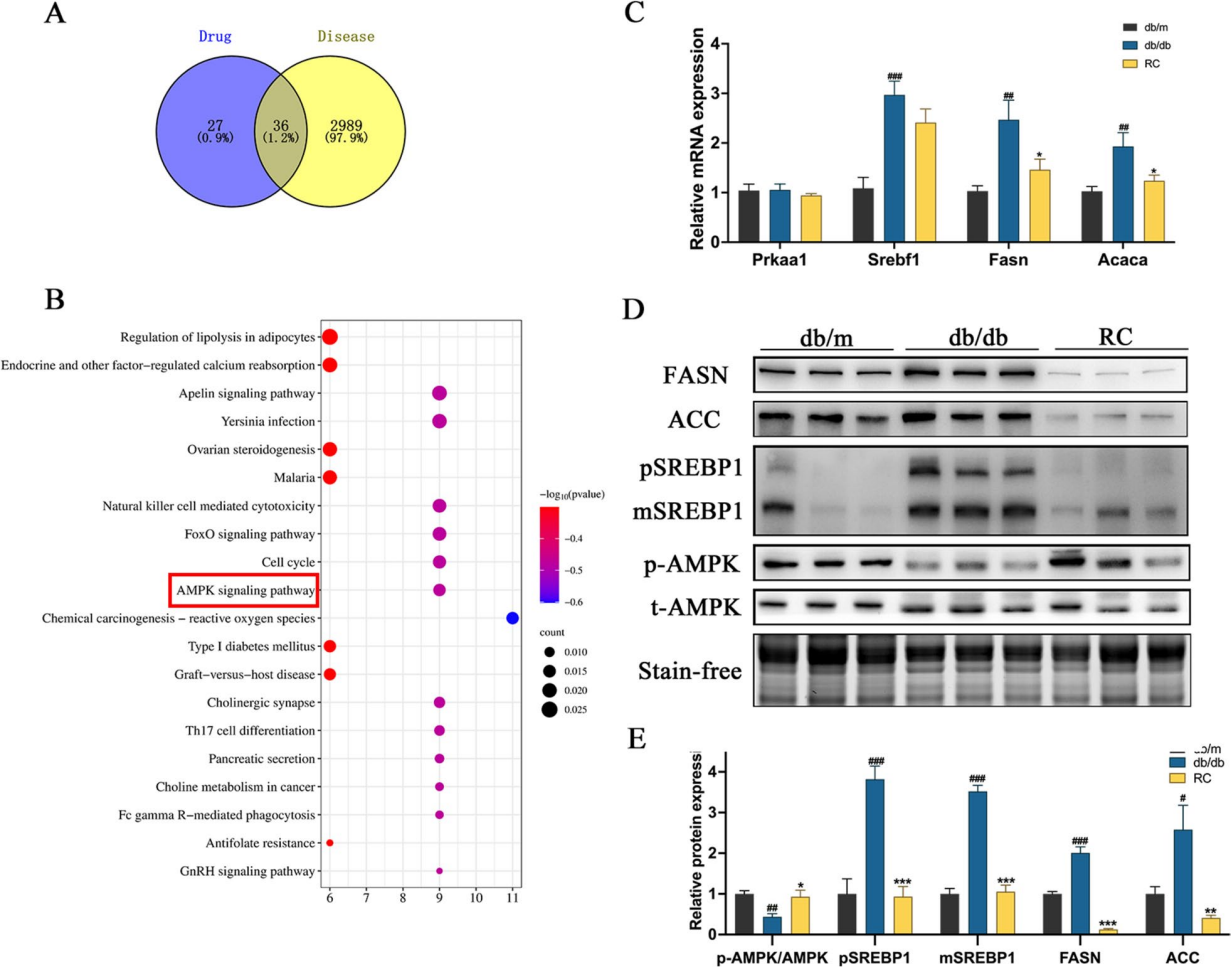
Predicting the AMPK Signaling Pathway Regulation through Network Pharmacology and Validating with Molecular Experiments. **A** Venn diagram of rhubarb and NAFLD-related targets. **B** KEGG pathway enrichment analysis. **C** Relative mRNA expression of *Prkaa1*, *Srebf1*, *Fasn* and *Acaca*. **D** Protein expressions of p-AMPK, t-AMPK, SREBP1 precursor (pSREBP1), mature SREBP1 (mSREBP1), FASN, and ACC in mice by Western blotting. **E** Quantitative analysis of the relative protein expression of p-AMPK, pSREBP1, mSREBP1, FASN, and ACC in liver. Stain-free technology was used as loading control. Uncropped stain-free gels of all samples used for total protein normalization are shown in Fig S3. The values reported in the figure represent the means ± SEM (*n* = 6). ###*P* < 0.001, ##*P* < 0.01, #*P* < 0.05 compared with the db/m group; ***P < 0.001, **P < 0.01, *P < 0.05 compared with the RC group

To elucidate the molecular mechanisms underlying RCS/SF scaffold's action against NAFLD, network pharmacology was employed for prediction (Fig. S1). KEGG Pathway analysis revealed 129 pathways associated with rhubarb's treatment of NAFLD (Fig. S2). The top 20 pathways are illustrated in Fig. 7B. The vital role of AMPK signalling in regulating the progression of NAFLD is widely recognized [27]. Therefore, subsequent molecular experiments were performed to affirm this connection. The qRT-PCR analysis showed a significant increase in the relative mRNA expression of *Srebf1*, *Acaca*, and *Fasn* in the livers of db/db mice, which was successfully suppressed by the RCS/SF scaffold. Meanwhile, there was no notable variation in the comparative mRNA expression of *prkaa1* among the three groups (Fig. 7C). Correspondingly, the RCS/SF scaffold significantly inhibited the levels of SREBP1, FASN, and ACC proteins, while enhancing the expression of p-AMPK (Fig. 7D, E). In summary, the findings indicate that the RCS/SF scaffold exerts alleviating hepatic lipid deposition effects by modulating the AMPK/SREBP1 pathway.

### Molecular docking verification

**Fig. 8:**
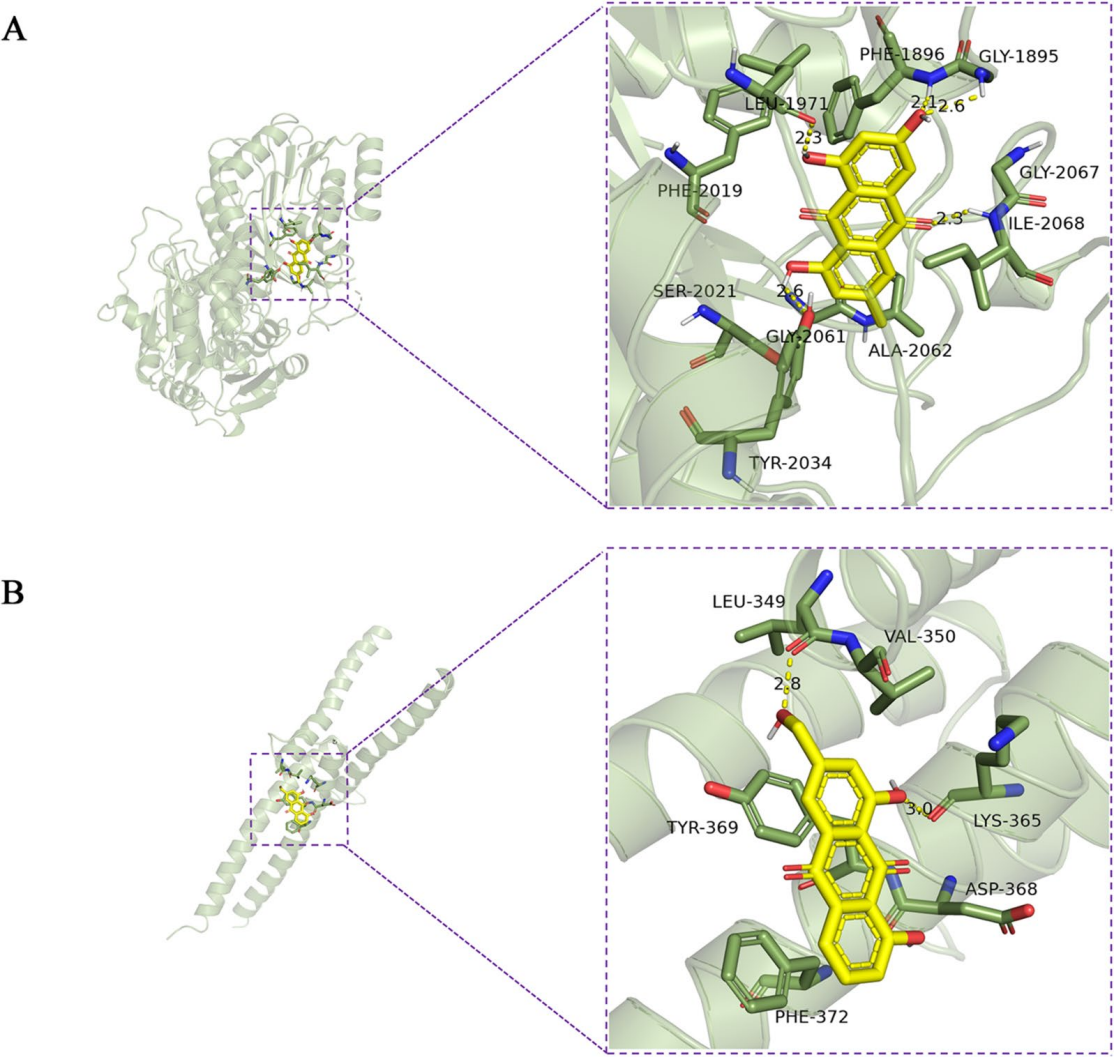
Molecular docking results. **A** Molecular docking results for emodin and FASN. **B** Molecular docking results for Aloe emodin and SREBP1

Based on the aforementioned analysis, the top five important targets were selected from the 22 active ingredients screened for rhubarb. These targets were chosen based on their higher degree values and will undergo semi-flexible docking. The details can be found in Tables S2 and S3. Affinity is employed to indicate the efficacy of a small molecule's binding to its target protein. A negative binding energy indicates that a small molecule can readily bind to its target protein, with a lower value indicating a higher probability of binding. The docking analysis demonstrated that emodin formed hydrogen bonds with specific amino acid residues (ILE-2068, GLY-1895, PHE-1896, LEU-1971, and GLY-2061) of the FASN protein. The calculated docking energy for this interaction was -9.5 kcal/mol (Fig. 8A). In addition, aloe-emodin established hydrogen bonds with LYS-365 and LEU-349 of SREBP1, resulting in a docking energy of -7.4 kcal/mol (Fig. 8B). These results indicate that the selected targets display a robust binding affinity with their corresponding active compounds, offering further proof of the reliability of the AMPK/SREBP1 pathway predictions made using network pharmacology, aligning with prior molecular experiments.

## Discussion

Studies conducted recently have showed that rhubarb contains several active compounds that exhibit various pharmacological effects, such as anti-inflammatory, anti-tumour, antioxidant, liver-protective, immune-regulating, lipid-lowering, and laxative properties [28–33]. Additionally, multiple animal experiments have demonstrated that rhubarb and its active components have a significant impact on lipid metabolism [34–36]. Specifically, they regulate this process by inhibiting lipogenesis, increasing lipolysis, and reducing lipid deposition. In a previous study, a biocompatible CS/SF scaffold was developed by incorporating rhubarb charcoal into a chitosan hydrogel scaffold, thereby demonstrating its antibacterial and anti-inflammatory efficacy [17]. The present research has demonstrated that the RCS/SF scaffold exerts an ameliorative impact on hepatic lipid deposition, inflammation, and oxidative stress in db/db mice. Accordingly, additional investigations were conducted in the current study to explore the potential impacts by examining the regulation of the AMPK/SREBP1 pathway, offering insights into the molecular mechanisms underlying these observed enhancements.

Db/db mice, characterised by mutations in the leptin receptor gene, exhibit a disrupted leptin signalling pathway, resulting in obesity, insulin resistance, hyperglycaemia, lipid metabolism disorders, inflammation, and oxidative stress. As a result, these mice are often employed as NAFLD research models for NAFLD [37]. The pathogenesis of NAFLD is complex and encompasses multiple factors. In addition to hepatic steatosis caused by the accumulation of hepatic triglycerides and de novo lipogenesis (DNL) [38], the liver can also accumulate toxic lipid species if it has difficulty processing primary metabolic energy substrates [39]. The accumulation of these substance causes stress, damage, and death in liver cells leading to inflammation and oxidative stress [40]. Ultimately, this sequence of occurrences has the potential to result in the formation of cirrhosis and hepatocellular carcinoma [41, 42].

AMPK, a serine/threonine protein kinase that is widely preserved, is essential for the regulation of cellular energy balance, maintenance of cellular energy equilibrium, and control of metabolism [43]. An increase in the intracellular ratio of Adenosine Monophosphate (AMP) to Adenosine Triphosphate (ATP) indicates a decrease in cellular energy levels, which leads to the activation of AMPK [44]. When activated, AMPK hinders the transcriptional function and decreases the SREBP1 expression. By inhibiting SREBP1, a key regulator of hepatic DNL, the transcriptional activities of FASN and ACC are subsequently reduced [45]. As a result, AMPK is considered a highly promising therapeutic target for NAFLD [46, 47]. Recent studies suggest that AMPK has the ability to rectify energy metabolism abnormalities within tumors. By orchestrating processes like the cell cycle, metabolism, and autophagy in cells, AMPK plays a pivotal role in combating both tumors and drug resistance [48–52]. Prior research has confirmed that emodin, a primary active compound found in rhubarb, acts as a regulator of AMPK [53]. The study demonstrated that emodin, by activating AMPK, led to a decrease in the levels of SREBP1 and FASN proteins in hepatocytes of rats that were given a high-fat diet (HFD). As expected, the study utilised network pharmacology predictions to determine that the AMPK signalling pathway is significantly involved in the therapeutic effects of rhubarb for NAFLD, as indicated by the KEGG enrichment results. Further experiments also verified that the use of RCS/SF scaffold effectively suppressed the expression of SREBP1, FASN, and ACC.

An imbalance in the cellular antioxidant homeostasis leads to oxidative stress, which is caused by the overproduction of reactive oxygen species (ROS) and reactive nitrogen species (RNS) within cells [54, 55]. As a result, crucial cellular elements like lipids, proteins, and deoxyribonucleic acid are susceptible to harm. The build-up of fats in liver cells in NAFLD results in the generation of detrimental lipid by-products like diacylglycerol, ceramides, cholesterol, and free fatty acids [56]. Concurrently, conditions such as insulin resistance, disorders in glucose metabolism, and abnormal cholesterol metabolism collectively disrupt the normal functioning of mitochondria, resulting in the increased production of ROS and the initiation of oxidative stress [57, 58]. During periods of oxidative stress, ROS directly target lipid molecules within the cell membrane, causing lipid peroxidation. This process results in the breakdown of lipid molecules and the creation of lipid peroxidation products, such as MDA. In addition, SOD converts superoxide radicals into hydrogen peroxide (H_2_O_2_), which is subsequently broken down by enzymes such as glutathione peroxidase (GPx) or CAT [59, 60]. When the organism undergoes oxidative stress, it triggers both enzymatic and non-enzymatic mechanisms to counteract the production of ROS [61]. Previous studies have confirmed that emodin has the ability to reduce hepatic oxidative stress in HFD-fed mice [62]. The results of the current research align with the findings, showing a significant decrease in MDA levels in the liver of db/db mice following treatment with RCS/SF scaffold, suggesting a decline in oxidative stress. Concurrently, the RCS/SF scaffold also facilitated the restoration of enzyme activity, specifically in CAT, SOD, and the NAD^+^/NADH ratio, suggesting a beneficial impact on antioxidant defence mechanisms and cellular energy metabolism.

Moreover, the release of inflammatory mediators exacerbates oxidative stress [63]. The process of oxidative stress initiates lipid peroxidation, which results in the liberation of pro-inflammatory agents, such as cytokines (IL-1β, IL-6, and TNF-α) and chemokines. These substances facilitate the recruitment and activation of immune cells in the liver [64]. The excessive build-up of lipids in the liver is closely connected to hepatocyte injury and the activation of Kupffer cells, which in turn triggers inflammatory responses that exacerbate hepatic disease [65]. Prior research has shown that emodin possesses the capacity to decrease the production of inflammatory factors in mice experiencing liver injury induced by lipopolysaccharide (LPS) [66]. The current investigation revealed that db/db mice displayed a significant rise in the mRNA expression levels of inflammatory factors (*Tnfα*, *Il-6*, and *Il-1β*) in comparison to db/m mice. Nevertheless, the RC group experienced a substantial decrease in these levels. Moreover, the immunohistochemical results further demonstrated a significant decrease in Tnfα protein expression following RCS/SF scaffold treatment in comparison to the db/db group.

In addition to the above, network pharmacology was utilized in the current study to predict the specific targets affected by rhubarb during the treatment of NAFLD. The analysis of KEGG enrichment indicated a notable association between the utilization of rhubarb for NAFLD and the activation of the AMPK signalling pathway. This was further validated from additional molecular docking studies, which confirmed the binding of five bioactive compounds in rhubarb with two downstream targets of AMPK, namely SREBP1 and FASN. Accordingly, the findings demonstrated that all five active components exhibited a strong binding activity with SREBP1 and FASN, as evidenced by their negative binding energy. Therefore, the combination of these two methods, along with molecular experiments, helps validate and establish a scientific foundation for comprehending the mechanism of the RCS/SF scaffold in addressing NAFLD.

**Fig. 9:**
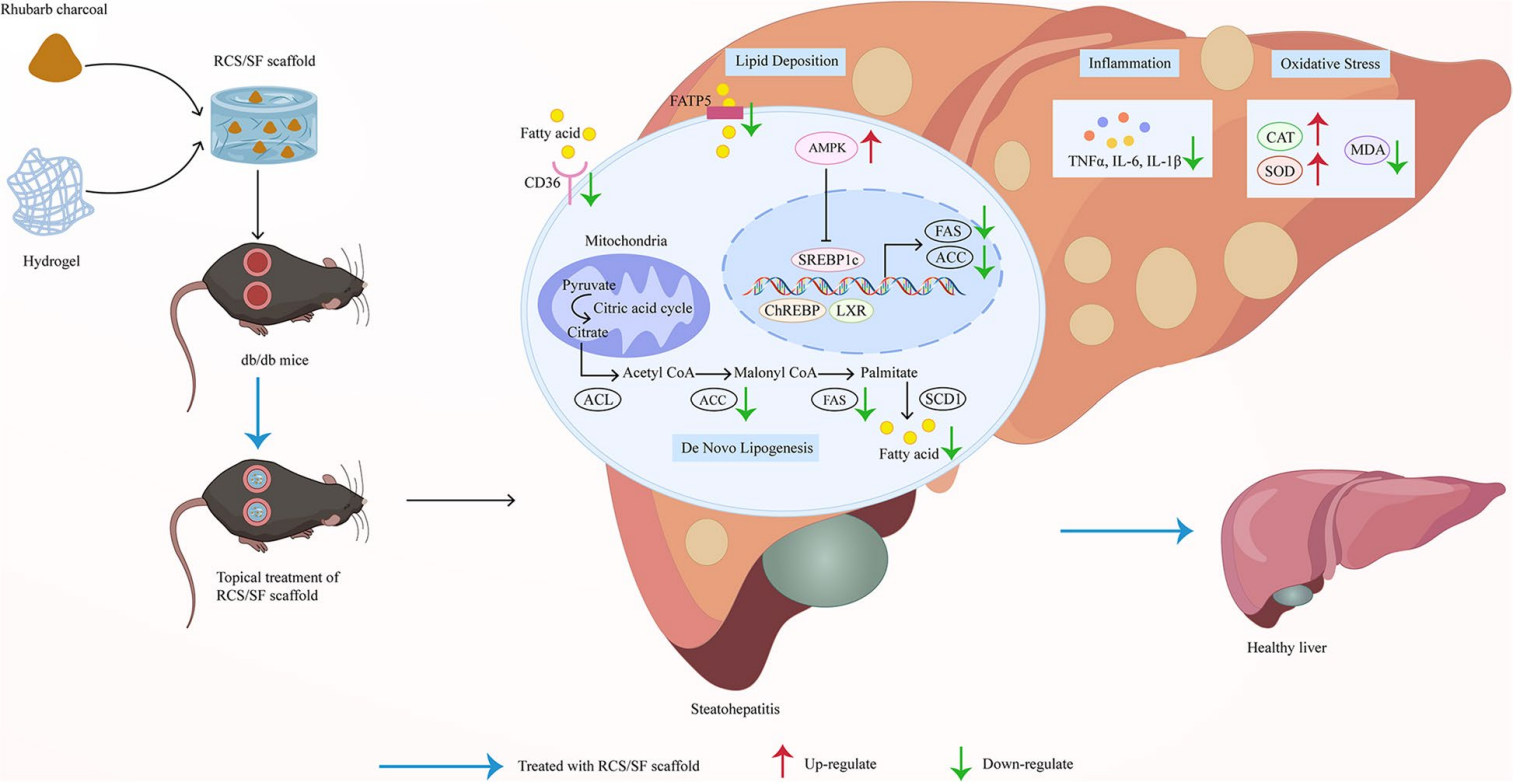
Schematic illustration showing partial mechanisms of the topical RCS/SF scaffold against NAFLD induced in db/db mice

According to the study findings, the RCS/SF scaffold applied topically has the ability to successfully decrease hepatic lipid buildup, inflammation, and oxidative stress by activating the AMPK signalling pathway, leading to an overall improvement in NAFLD in db/db mice (Fig. 9). Moreover, the application of the "external and internal reshaping" theory elucidates the capability of the RCS/SF scaffold to have a therapeutic effect that targets multiple aspects and dimensions. To summarise, the hypothesis was that the RCS/SF scaffold not only had the potential to serve as an antimicrobial dressing for the treatment of diabetic wounds but also had a positive impact on alleviating hepatic lipid deposition effects by regulating lipid metabolism. As observed, the initial scientific evidence not only supports this hypothesis but also advocates the potential use of the RCS/SF scaffold for use in clinical applications.

### Study strength and limitations

This study reveals the effectiveness of applying RCS/SF scaffolds in significantly reducing hepatic lipid deposition in db/db mice for the first time. The mechanism seems closely linked to regulating the AMPK signalling pathway. The research also preliminary explores the scaffold's impact on inflammation, oxidative stress, and the regulation of genes related to glycolipid metabolism, aligning with the "external and internal reshaping" theory. However, the study has several limitations, and more in-depth research is needed to explain the specific mechanisms involved. Additionally, exploring the impact of RCS/SF scaffold on skeletal muscle or other metabolism-related organs is worthy of further investigation.

## Conclusions

In conclusion, this study elucidates that the topical application of RCS/SF scaffold effectively mitigates inflammation, oxidative stress, and hepatic lipid deposition in db/db mice by modulating the AMPK signalling pathway. This lays the groundwork for its potential therapeutic application in managing metabolic disorders associated with diabetic wound healing. Additionally, preceding investigations have underscored the superior wound healing properties of RCS/SF scaffold. Consequently, this research establishes a robust theoretical framework for the clinical implementation of RCS/SF scaffold as an innovative dressing for diabetic wounds complicated by metabolic issues.

### Supplementary Information

**Additional file 1: Table S1.** Primers used in RT-qPCR analysis.** Table S2.** 22 Potential active ingredients of Rhubarb.** Table S3.** Molecular docking results of the five main active ingredients with FASN and SREBP1.** Fig S1.** "Drug-component-target" prediction and Protein-Protein Interaction Network (PPI) construction.(A)"Drug-component-target" prediction results (rectangles represent targets, ellipses represent components, and diamond nodes represent drugs), (B) 36 Intersection target genes of Rhubarb and NAFLD, (C) The core targets of PPI.** Fig S2.** Biological function enrichment analysis. Top ten Gene Ontology terms for biological processes (BP), (B)Top ten Gene Ontology terms for cellular component (CC), (C)Top ten Gene Ontology terms for molecular function (MF), (D) GO results of three ontologies.** Fig S3.** Uncropped stain-free gels of all samples used for total protein normalization.** Fig S4.** A certificate of language editing.** Fig S5.** All the raw data for uncorrupted western blotting.

**Additional file 2.**

## Data Availability

The data supporting the outcomes of this investigation are accessible from the corresponding author upon request.

## Abbreviations

ACC: Acetyl CoA carboxylase
Acly: ATP citrate lyase
ALT: Alanine aminotransferase
AMP: Adenosine Monophosphate
AMPK: Adenosine monophosphate-activated protein kinase
AST: Aspartate aminotransferase
ATP: Adenosine Triphosphate
BSA: Bovine serum albumin
CAT: Catalase
CD36: CD36 molecule
cDNA: Complementary deoxyribonucleic acid
Dgat2: Diacylglycerol O-acyltransferase 2
DL: Drug-likeness
DNL: De novo lipogenesis
DUs: Diabetic ulcers
FASN: Fatty acid synthase
GO: Gene ontology
GPx: Glutathione peroxidase
H&E: Haematoxylin and eosin
H_2_O_2_: Hydrogen peroxide
HDL-C: High-density lipoprotein cholesterol
HFD: High-fat diet
Il-10: Interleukin 10
Il-1β: Interleukin 1 beta
Il-6: Interleukin 6
KEGG: Kyoto encyclopaedia of genes and genomes
LDL-C: Low-density lipoprotein cholesterol
LPS: Lipopolysaccharide
MDA: Malondialdehyde
Mgat2: Mannoside acetylglucosaminyltransferase 2
Mlxipl: MLX interacting protein-like
NAFLD: Non-alcoholic fatty liver disease
NAS: NAFLD activity score
NASH: Non-alcoholic steatohepatitis
Nr1h3: Nuclear receptor subfamily 1 group h member 3
PAS: Periodic acid-Schiff
Pck1: Phosphoenolpyruvate carboxykinase 1
Pklr: Pyruvate kinase liver and red blood cell
qRT-PCR: Quantitative real-time PCR
RNS: Reactive nitrogen species
ROS: Reactive oxygen species
Rplp0: Ribosomal protein lateral stalk subunit P0
RSC/SF: Rhubarb charcoal-crosslinked chitosan/silk fibroin sponge
Slc27a4: Solute carrier family 27 member 4
Slc27a5: Solute carrier family 27 member 5
Slc2a2: Solute carrier family 2 member 2
SOD: Superoxide dismutase
SREBP1: Sterol regulatory element binding protein 1
T2DM: Type 2 diabetes mellitus
TBST: Tris buffered saline with tween
TC: Total cholesterol
TCM: Traditional Chinese medicine
TCMSP: Traditional Chinese Medicine Database and Analysis Platform
TG: Triglyceride
Tnfα: Tumour necrosis factor alpha

## References

[1] Younossi Z, Tacke F, Arrese M, Chander Sharma B, Mostafa I, Bugianesi E (2019). Global perspectives on nonalcoholic fatty liver disease and nonalcoholic Steatohepatitis. Hepatology.

[2] Mansouri A, Gattolliat C-H, Asselah T (2018). Mitochondrial dysfunction and signaling in chronic liver diseases. Gastroenterology.

[3] Powell EE, Wong VW-S, Rinella M (2021). Non-alcoholic fatty liver disease. Lancet.

[4] Huang DQ, El-Serag HB, Loomba R (2021). Global epidemiology of NAFLD-related HCC: trends, predictions, risk factors and prevention. Nat Reviews Gastroenterol Hepatol.

[5] Tilg H, Moschen AR (2010). Evolution of inflammation in nonalcoholic fatty liver disease: the multiple parallel hits hypothesis. Hepatology.

[6] Allen AM, Lazarus JV, Younossi ZM (2023). Healthcare and socioeconomic costs of NAFLD: a global framework to navigate the uncertainties. J Hepatol.

[7] McDermott K, Fang M, Boulton AJM, Selvin E, Hicks CW (2023). Etiology, epidemiology, and disparities in the burden of diabetic foot ulcers. Diabetes Care.

[8] Bardill JR, Laughter MR, Stager M, Liechty KW, Krebs MD, Zgheib C (2022). Topical gel-based biomaterials for the treatment of diabetic foot ulcers. Acta Biomater.

[9] Sethuram L, Thomas J, Mukherjee A, Chandrasekaran N (2022). A review on contemporary nanomaterial-based therapeutics for the treatment of diabetic foot ulcers (DFUs) with special reference to the Indian scenario. Nanoscale Adv.

[10] Liu Y, Li Y, Du Y, Huang T, Zhu C (2020). Multicenter clinical trials analyzing efficacy and safety of topical cortex phellodendri compound fluid in treatment of diabetic foot ulcers. Med Sci Monit.

[11] Zhong L, Shi C, Hou Q, Yang R, Li M, Fu X (2022). Promotive effects of four herbal medicine ARCC on wound healing in mice and human. Health Sci Rep.

[12] Li S, Zhao J, Liu J, Xiang F, Lu D, Liu B (2011). Prospective randomized controlled study of a Chinese herbal medicine compound Tangzu Yuyang ointment for chronic diabetic foot ulcers: a preliminary report. J Ethnopharmacol.

[13] Fei J, Ling YM, Zeng MJ, Zhang KW (2019). Shixiang plaster, a traditional Chinese medicine, promotes healing in a rat model of diabetic ulcer through the Receptor for Advanced Glycation End products (RAGE)/Nuclear factor kappa B (NF-kappaB) and vascular endothelial growth factor (VEGF)/Vascular cell adhesion molecule-1 (VCAM-1)/Endothelial nitric oxide synthase (eNOS) signaling pathways. Med Sci Monit.

[14] Jia H, Yang B, Li Y, Liang C, Lu H, Lin D (2018). Chinese medicine ulcer oil promotes the healing of diabetic foot ulcers. J Int Med Res.

[15] Soares RDF, Campos MGN, Ribeiro GP, Salles BCC, Cardoso NS, Ribeiro JR (2020). Development of a chitosan hydrogel containing flavonoids extracted from Passiflora edulis leaves and the evaluation of its antioxidant and wound healing properties for the treatment of skin lesions in diabetic mice. J Biomed Mater Res A.

[16] Yang H, Song L, Sun B, Chu D, Yang L, Li M (2021). Modulation of macrophages by a paeoniflorin-loaded hyaluronic acid-based hydrogel promotes diabetic wound healing. Mater Today Bio.

[17] Wang S, Zhang Y, Shi Y, He Q, Tan Q, Peng Z (2023). Rhubarb charcoal-crosslinked chitosan/silk fibroin sponge scaffold with efficient hemostasis, inflammation, and angiogenesis for promoting diabetic wound healing. Int J Biol Macromol.

[18] Chen Y, Chen N, Feng X (2021). The role of internal and external stimuli in the rational design of skin-specific drug delivery systems. Int J Pharm.

[19] Xu L, Zang D, Li H, Sulitang A, Li Y, Ma J (2022). Five traditional Chinese medicine external treatment methods combined with Mecobalamin for diabetic peripheral neuropathy: a network meta-analysis. Evid Based Complement Alternat Med.

[20] Jobbins AM, Yu S, Paterson HAB, Maude H, Kefala-Stavridi A, Speck C (2023). Pre-RNA splicing in metabolic homeostasis and liver disease. Trends Endocrinol Metab.

[21] Régnier M, Carbinatti T, Parlati L, Benhamed F, Postic C (2023). The role of ChREBP in carbohydrate sensing and NAFLD development. Nat Rev Endocrinol.

[22] Shi Y, Wang S, Zhang W, Zhu Y, Fan Z, Huang Y (2022). Bone marrow mesenchymal stem cells facilitate diabetic wound healing through the restoration of epidermal cell autophagy via the HIF-1α/TGF-β1/SMAD pathway. Stem Cell Res Ther.

[23] Kleiner DE, Brunt EM, Van Natta M, Behling C, Contos MJ, Cummings OW (2005). Design and validation of a histological scoring system for nonalcoholic fatty liver disease. Hepatology.

[24] Liu Y, Li D, Wang S, Peng Z, Tan Q, He Q (2023). 6-Gingerol ameliorates hepatic Steatosis, inflammation and oxidative stress in high-fat diet-fed mice through activating LKB1/AMPK signaling. Int J Mol Sci.

[25] Lessard SJ, MacDonald TL, Pathak P, Han MS, Coffey VG, Edge J (2018). JNK regulates muscle remodeling via myostatin/SMAD inhibition. Nat Commun.

[26] Park JS, Rustamov N, Roh YS (2023). The roles of NFR2-regulated oxidative stress and mitochondrial quality control in chronic liver diseases. Antioxid (Basel).

[27] Hardie DG (2011). AMP-activated protein kinase: an energy sensor that regulates all aspects of cell function. Genes Dev.

[28] Lee EH, Baek SY, Park JY, Kim YW (2020). Emodin in Rheum Undulatum inhibits oxidative stress in the liver via AMPK with Hippo/Yap signalling pathway. Pharm Biol.

[29] Wang Y, Zhang J, Xu Z, Zhang G, Lv H, Wang X (2022). Identification and action mechanism of lipid regulating components from Rhei Radix et rhizoma. J Ethnopharmacol.

[30] Ye B, Chen X, Dai S, Han J, Liang X, Lin S (2019). Emodin alleviates myocardial ischemia/reperfusion injury by inhibiting gasdermin D-mediated pyroptosis in cardiomyocytes. Drug Des Devel Ther.

[31] Stompor-Gorący M (2021). The Health benefits of Emodin, a natural anthraquinone derived from Rhubarb-A summary update. Int J Mol Sci.

[32] Brkanac SR, Gerić M, Gajski G, Vujčić V, Garaj-Vrhovac V, Kremer D (2015). Toxicity and antioxidant capacity of Frangula alnus Mill. Bark and its active component emodin. Regul Toxicol Pharmacol.

[33] Yang HY, Wu J, Lu H, Cheng ML, Wang BH, Zhu HL (2023). Emodin suppresses oxaliplatin-induced neuropathic pain by inhibiting COX2/NF-κB mediated spinal inflammation. J Biochem Mol Toxicol.

[34] Cao Y, Chang S, Dong J, Zhu S, Zheng X, Li J (2016). Emodin ameliorates high-fat-diet induced insulin resistance in rats by reducing lipid accumulation in skeletal muscle. Eur J Pharmacol.

[35] Fang JY, Huang TH, Chen WJ, Aljuffali IA, Hsu CY (2022). Rhubarb hydroxyanthraquinones act as antiobesity agents to inhibit adipogenesis and enhance lipolysis. Biomed Pharmacother.

[36] Yu L, Gong L, Wang C, Hu N, Tang Y, Zheng L (2020). Radix Polygoni Multiflori and its main component Emodin Attenuate non-alcoholic fatty liver disease in zebrafish by regulation of AMPK signaling pathway. Drug Des Devel Ther.

[37] Ibrahim SH, Hirsova P, Malhi H, Gores GJ (2016). Animal models of nonalcoholic steatohepatitis: eat, delete, and inflame. Dig Dis Sci.

[38] Esler WP, Cohen DE. Pharmacologic inhibition of lipogenesis for the treatment of NAFLD. J Hepatol. 2024;80(2):362–77. 10.1016/j.jhep.2023.10.042 PMC10842769 37977245

[39] Gebru YA, Gupta H, Kim HS, Eom JA, Kwon GH, Park E (2021). T cell subsets and natural killer cells in the pathogenesis of nonalcoholic fatty liver disease. Int J Mol Sci.

[40] Geier A, Tiniakos D, Denk H, Trauner M (2021). From the origin of NASH to the future of metabolic fatty liver disease. Gut.

[41] Boursier J, Mueller O, Barret M, Machado M, Fizanne L, Araujo-Perez F (2016). The severity of nonalcoholic fatty liver disease is associated with gut dysbiosis and shift in the metabolic function of the gut microbiota. Hepatology.

[42] Velliou R-I, Legaki A-I, Nikolakopoulou P, Vlachogiannis NI, Chatzigeorgiou A (2023). Liver endothelial cells in NAFLD and transition to NASH and HCC. Cell Mol Life Sci.

[43] Herzig S, Shaw RJ (2018). AMPK: guardian of metabolism and mitochondrial homeostasis. Nat Rev Mol Cell Biol.

[44] Trefts E, Shaw RJ (2021). AMPK: restoring metabolic homeostasis over space and time. Mol Cell.

[45] Su F, Koeberle A. Regulation and targeting of SREBP-1 in hepatocellular carcinoma. Cancer Metastasis Rev. 2023. 10.1007/s10555-023-10156-5. Epub ahead of print. 10.1007/s10555-023-10156-5 PMC11156753 38036934

[46] Peng Y, Qi Z, Xu Y, Yang X, Cui Y, Sun Q (2024). AMPK and metabolic disorders: the opposite roles of dietary bioactive components and food contaminants. Food Chem.

[47] Hsu C-C, Peng D, Cai Z, Lin H-K (2022). AMPK signaling and its targeting in cancer progression and treatment. Semin Cancer Biol.

[48] Li B, Chen Q, Feng Y, Wei T, Zhong Y, Zhang Y (2023). Glucose restriction induces AMPK-SIRT1-mediated circadian clock gene per expression and delays NSCLC progression. Cancer Lett.

[49] Qin Y, Ashrafizadeh M, Mongiardini V, Grimaldi B, Crea F, Rietdorf K (2023). Autophagy and cancer drug resistance in dialogue: pre-clinical and clinical evidence. Cancer Lett.

[50] Sun T, Liu B, Cao Y, Li Y, Cai L, Yang W (2024). AMPK-mediated CD47 H3K4 methylation promotes phagocytosis evasion of glioma stem cells post-radiotherapy. Cancer Lett.

[51] Wang Z, Wang Y, Shen N, Liu Y, Xu X, Zhu R (2024). AMPKα1-mediated ZDHHC8 phosphorylation promotes the palmitoylation of SLC7A11 to facilitate ferroptosis resistance in glioblastoma. Cancer Lett.

[52] Sui Q, Hu Z, Liang J, Lu T, Bian Y, Jin X (2024). Targeting TAM-secreted S100A9 effectively enhances the tumor-suppressive effect of metformin in treating lung adenocarcinoma. Cancer Lett.

[53] Li W, Wang D, Li M, Li B (2021). Emodin inhibits the proliferation of papillary thyroid carcinoma by activating AMPK. Exp Ther Med.

[54] Arfin S, Jha NK, Jha SK, Kesari KK, Ruokolainen J, Roychoudhury S (2021). Oxidative stress in Cancer Cell Metabolism. Antioxid (Basel).

[55] Juan CA, de la Pérez JM, Plou FJ, Pérez-Lebeña E (2021). The Chemistry of reactive oxygen species (ROS) revisited: outlining their role in Biological macromolecules (DNA, lipids and proteins) and Induced pathologies. Int J Mol Sci.

[56] Seen S (2021). Chronic liver disease and oxidative stress - a narrative review. Expert Rev Gastroenterol Hepatol.

[57] Rolo AP, Teodoro JS, Palmeira CM (2012). Role of oxidative stress in the pathogenesis of nonalcoholic steatohepatitis. Free Radic Biol Med.

[58] Spahis S, Delvin E, Borys J-M, Levy E (2017). Oxidative stress as a critical factor in nonalcoholic fatty liver disease Pathogenesis. Antioxid Redox Signal.

[59] Ore A, Akinloye OA (2019). Oxidative stress and antioxidant biomarkers in clinical and experimental models of non-alcoholic fatty liver disease. Med (Kaunas Lithuania).

[60] Feng G, Byrne CD, Targher G, Wang F, Zheng M-H (2022). Ferroptosis and metabolic dysfunction-associated fatty liver disease: is there a link?. Liver Int.

[61] Arroyave-Ospina JC, Wu Z, Geng Y, Moshage H (2021). Role of oxidative stress in the pathogenesis of non-alcoholic fatty liver disease: implications for prevention and therapy. Antioxid (Basel).

[62] Shen C, Pan Z, Wu S, Zheng M, Zhong C, Xin X (2021). Emodin palliates high-fat diet-induced nonalcoholic fatty liver disease in mice via activating the farnesoid X receptor pathway. J Ethnopharmacol.

[63] Verma AK, Sharma A, Subramaniyam N, Gandhi CR (2022). Augmenter of liver regeneration: mitochondrial function and steatohepatitis. J Hepatol.

[64] de Souza Teixeira AA, Souza CO, Biondo LA, Sanches Silveira L, Lima EA, Batatinha HA (2018). Short-term treatment with metformin reduces hepatic lipid accumulation but induces liver inflammation in obese mice. Inflammopharmacology.

[65] Cusi K (2012). Role of obesity and lipotoxicity in the development of nonalcoholic steatohepatitis: pathophysiology and clinical implications. Gastroenterology.

[66] Xiao D, Hu Y, Fu Y, Wang R, Zhang H, Li M (2019). Emodin improves glucose metabolism by targeting microRNA-20b in insulin-resistant skeletal muscle. Phytomedicine: Int J Phytotherapy Phytopharmacology.

