# Supplementary material for: Topical rhubarb charcoal-crosslinked chitosan/silk fibroin sponge scaffold for the repair of diabetic ulcers improves hepatic lipid deposition in db/db mice via the AMPK signalling pathway
Source: Lipids Health Dis. 2024 Feb 20;23:52. doi: 10.1186/s12944-024-02041-z (PMC10877747; doi:10.1186/s12944-024-02041-z)
Supplement: Supplementary file 1 — Additional file 1: Table S1. Primers used in RT-qPCR analysis. Table S2. 22 Potential active ingredients of Rhubarb. Table S3. Molecular docking results of the five main active ingredients with FASN and SREBP1. Fig S1. "Drug-component-target" prediction and Protein-Protein Interaction Network (PPI) construction.(A)"Drug-component-target" prediction results (rectangles represent targets, ellipses represent components, and diamond nodes represent drugs), (B) 36 Intersection target genes of Rhubarb and NAFLD, (C) The core targets of PPI. Fig S2. Biological function enrichment analysis. Top ten Gene Ontology terms for biological processes (BP), (B)Top ten Gene Ontology terms for cellular component (CC), (C)Top ten Gene Ontology terms for molecular function (MF), (D) GO results of three ontologies. Fig S3. Uncropped stain-free gels of all samples used for total protein normalization. Fig S4. A certificate of language editing. Fig S5. All the raw data for uncorrupted western blotting. [file 12944_2024_2041_MOESM1_ESM.docx]

**Supplementary Materials for**

Topical rhubarb charcoal-crosslinked chitosan/silk fibroin sponge scaffold for diabetic ulcers repair improves hepatic lipid deposition in db/db mice via AMPK signaling pathway

Table S1. Primers used in RT-qPCR analysis.

| Gene Forward prime Reverse primer |
| --- |
| \| *Mlxipl* \| AGATGGAGAACCGACGTATCA \| ACTGAGCGTGCTGACAAGTC \| \| --- \| --- \| --- \| \| *Pklr* \| CTTGCTCTACCGTGAGCCTC \| ACCACAATCACCAGATCACC \| \| *Nr1h3* \| ACAGAGCTTCGTCCACAAAAG \| GCGTGCTCCCTTGATGACA \| \| *Acly* \| ACCCTTTCACTGGGGATCACA \| GACAGGGATCAGGATTTCCTTG \| \| *Cd36* \| ATGGGCTGTGATCGGAACTG \| GTCTTCCCAATAAGCATGTCTCC \| \| *Slc27a4* \| ACTGTTCTCCAAGCTAGTGCT \| GATGAAGACCCGGATGAAACG \| \| *Slc27a5* \| CTACGCTGGCTGCATATAGATG \| CCACAAAGGTCTCTGGAGGAT \| \| *Dgat2* \| GCGCTACTTCCGAGACTACTT \| GGGCCTTATGCCAGGAAACT \| \| *Mgat2* \| ATGAGGTTCCGCATCTACAAAC \| GCCCATTGCTACTCCAGAGG \| \| *Slc2a2* \| ATCGCTCCAACCACACTCAG \| GCTGAGGCCAGCAATCTGAC \| \| *Pck1* \| CTGCATAACGGTCTGGACTTC \| CAGCAACTGCCCGTACTCC \| \| *G6pc1* \| CGACTCGCTATCTCCAAGTGA \| GTTGAACCAGTCTCCGACCA \| \| *Tnfα* \| CCCTCACACTCAGATCATCTTCT \| GCTACGACGTGGGCTACAG \| \| *Il 1b* \| GCAACTGTTCCTGAACTCAACT \| ATCTTTTGGGGTCCGTCAACT \| \| *Il 10* \| CTTACTGACTGGCATGAGGATCA \| GCAGCTCTAGGAGCATGTGG \| \| *Il 6* \| TAGTCCTTCCTACCCCAATTTCC \| TTGGTCCTTAGCCACTCCTTC \| \| *Srebf1* \| TGACCCGGCTATTCCGTGA \| CTGGGCTGAGCAATACAGTTC \| \| *Prkaa1* \| GTCAAAGCCGACCCAATGATA \| CGTACACGCAAATAATAGGGGTT \| \| Fasn \| AGGTGGTGATAGCCGGTATGT \| TGGGTAATCCATAGAGCCCAG \| \| *Acaca* \| CTCCCGATTCATAATTGGGTCTG \| TCGACCTTGTTTTACTAGGTGC \| |

Table S2. 22 Potential active ingredients of Rhubarb.

| MOL ID | Chemical compound | DL |
| --- | --- | --- |
| MOL001729 | Crysophanol | 0.21 |
| MOL001986 | β-sitosterol | 0.71 |
| MOL002230 | (+)-Catechin-pentaacetate | 0.77 |
| MOL002231 | (-)-Epicatechin-pentaacetate | 0.77 |
| MOL002235 | EUPATIN | 0.41 |
| MOL002238 | 3-Hydroxy-25-norfriedel-3,1(10)-dien-2-one-30-oic acid | 0.78 |
| MOL002244 | Chrysophanol glucoside | 0.76 |
| MOL002249 | gallocatechin | 0.27 |
| MOL002256 | 1,8-dihydroxy-3-methoxy-2,6-dimethyl-9,10-anthraquinone | 0.29 |
| MOL002258 | Physcion-9-O-beta-D-glucopyranoside | 0.3 |
| MOL002261 | ZINC04081604 | 0.44 |
| MOL002268 | rhein | 0.28 |
| MOL002281 | Toralactone | 0.24 |
| MOL002286 | laccaic acid D | 0.31 |
| MOL002297 | Daucosterol_qt | 0.7 |
| MOL002300 | 10beta-Hydroxy-6beta-isobutyrylfuranoeremophilane | 0.29 |
| MOL002302 | RHAPONTIN | 0.55 |
| MOL000358 | beta-sitosterol | 0.75 |
| MOL000471 | aloe-emodin | 0.24 |
| MOL000472 | emodin | 0.24 |
| MOL000476 | Physcion | 0.27 |
| MOL000096 | (-)-catechin | 0.24 |

Table S3. Molecular docking results of the five main active ingredients with FASN and SREBP1.

| Target | PDB ID | Chemical compound | Binding energy（kcal/mol） |
| --- | --- | --- | --- |
| FASN | 6NNA | aloe-emodin | -9 |
|  |  | beta-sitosterol | -9.4 |
|  |  | catechin | -8.9 |
|  |  | emodin | -9.5 |
|  |  | Eupatin | -9.2 |
| SREBP1 | 1AM9 | aloe-emodin | -7.4 |
|  |  | beta-sitosterol | -7.3 |
|  |  | catechin | -6.4 |
|  |  | emodin | -7.2 |
|  |  | Eupatin | -6.3 |

**
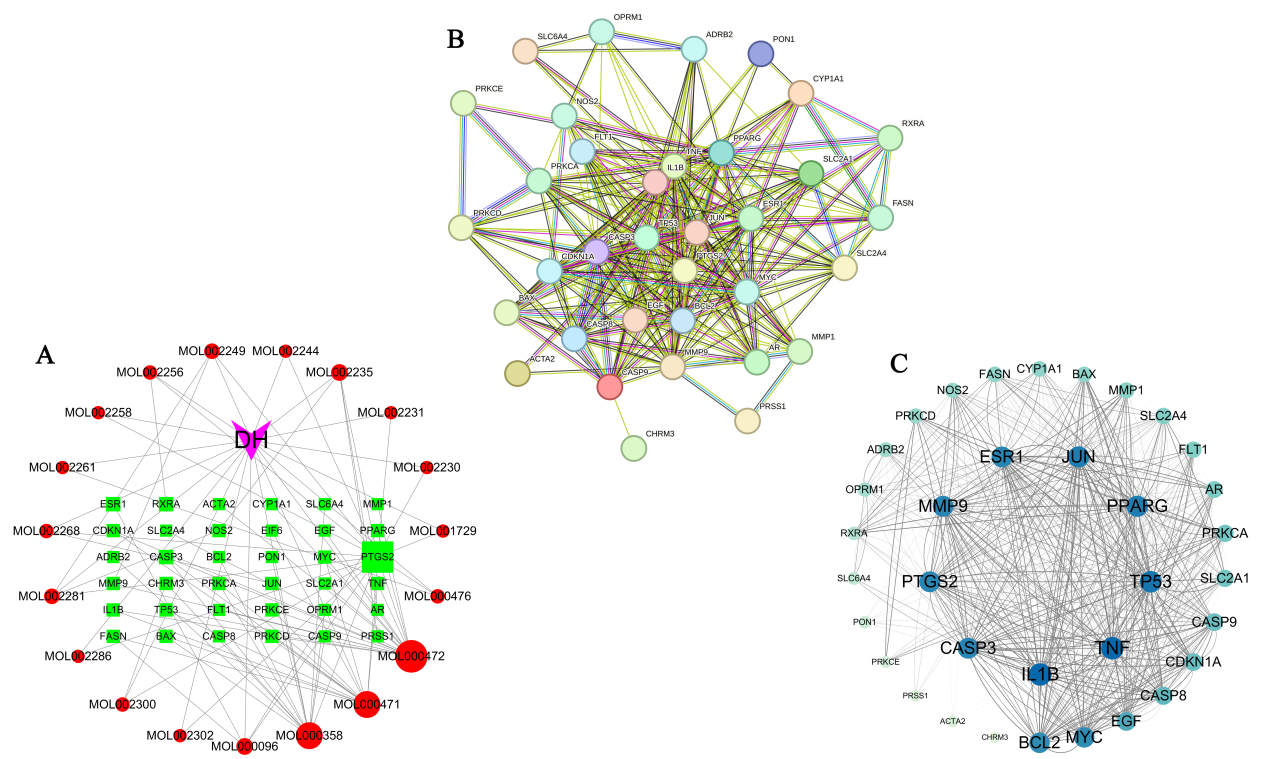
**

Fig S1. "Drug-component-target" prediction and Protein-Protein Interaction Network (PPI) construction.(A)"Drug-component-target" prediction results (rectangles represent targets, ellipses represent components, and diamond nodes represent drugs), (B) 36 Intersection target genes of Rhubarb and NAFLD, (C) The core targets of PPI.

**
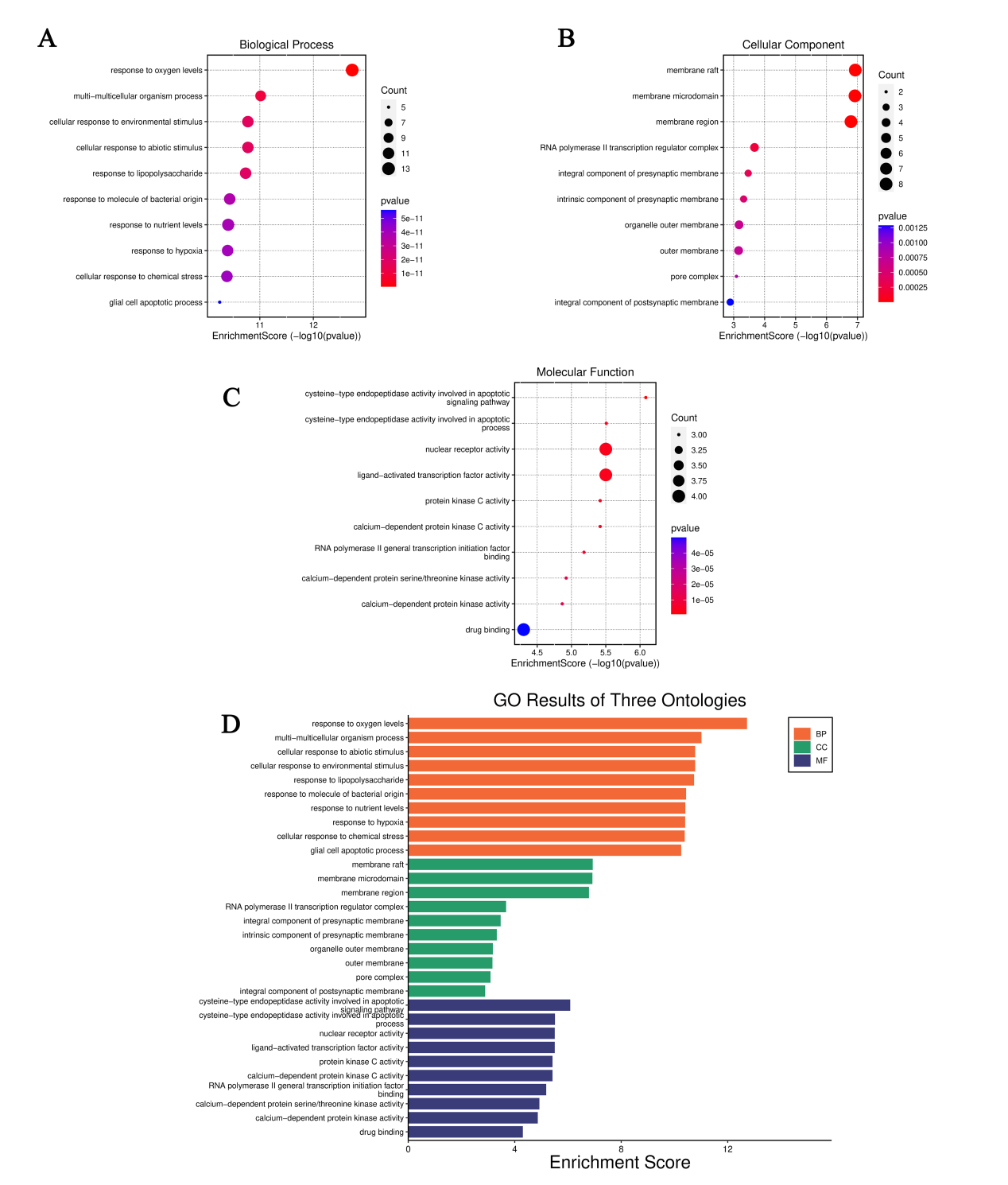
**

Fig S2. Biological function enrichment analysis.

1. Top ten Gene Ontology terms for biological processes (BP), (B)Top ten Gene Ontology terms for cellular component (CC), (C)Top ten Gene Ontology terms for molecular function (MF), (D) GO results of three ontologies.

**
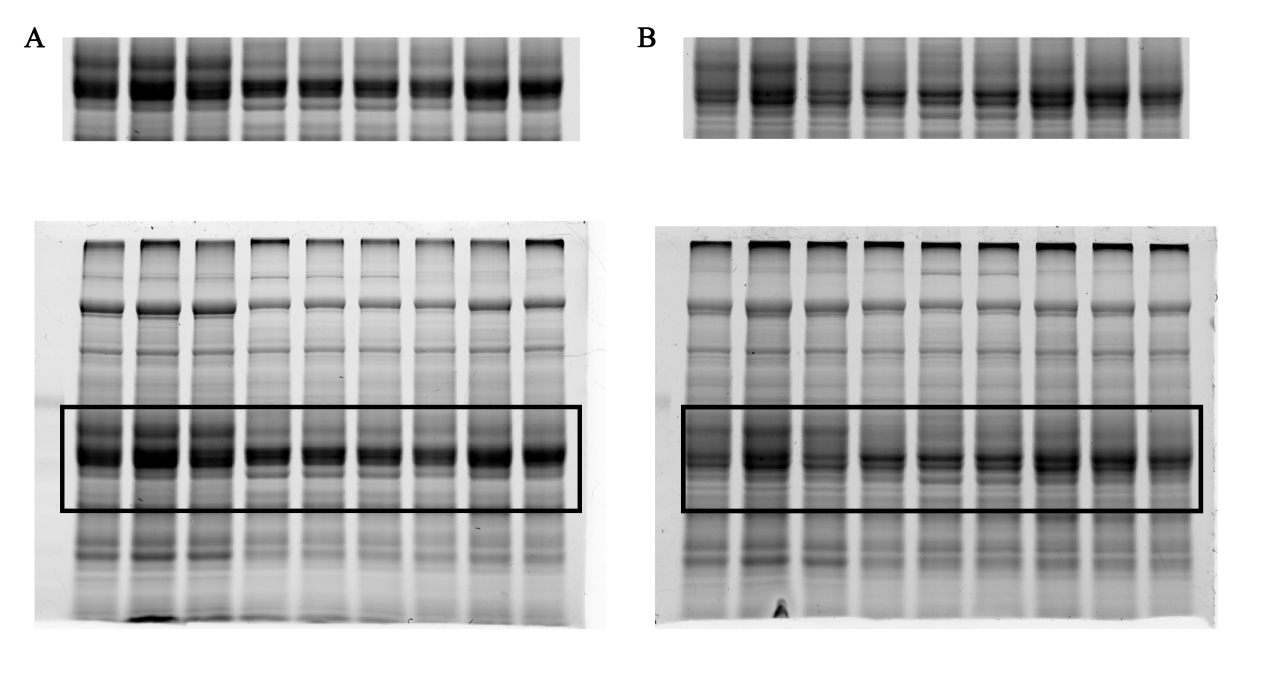
**

Fig S3. Uncropped stain-free gels of all samples used for total protein normalization.


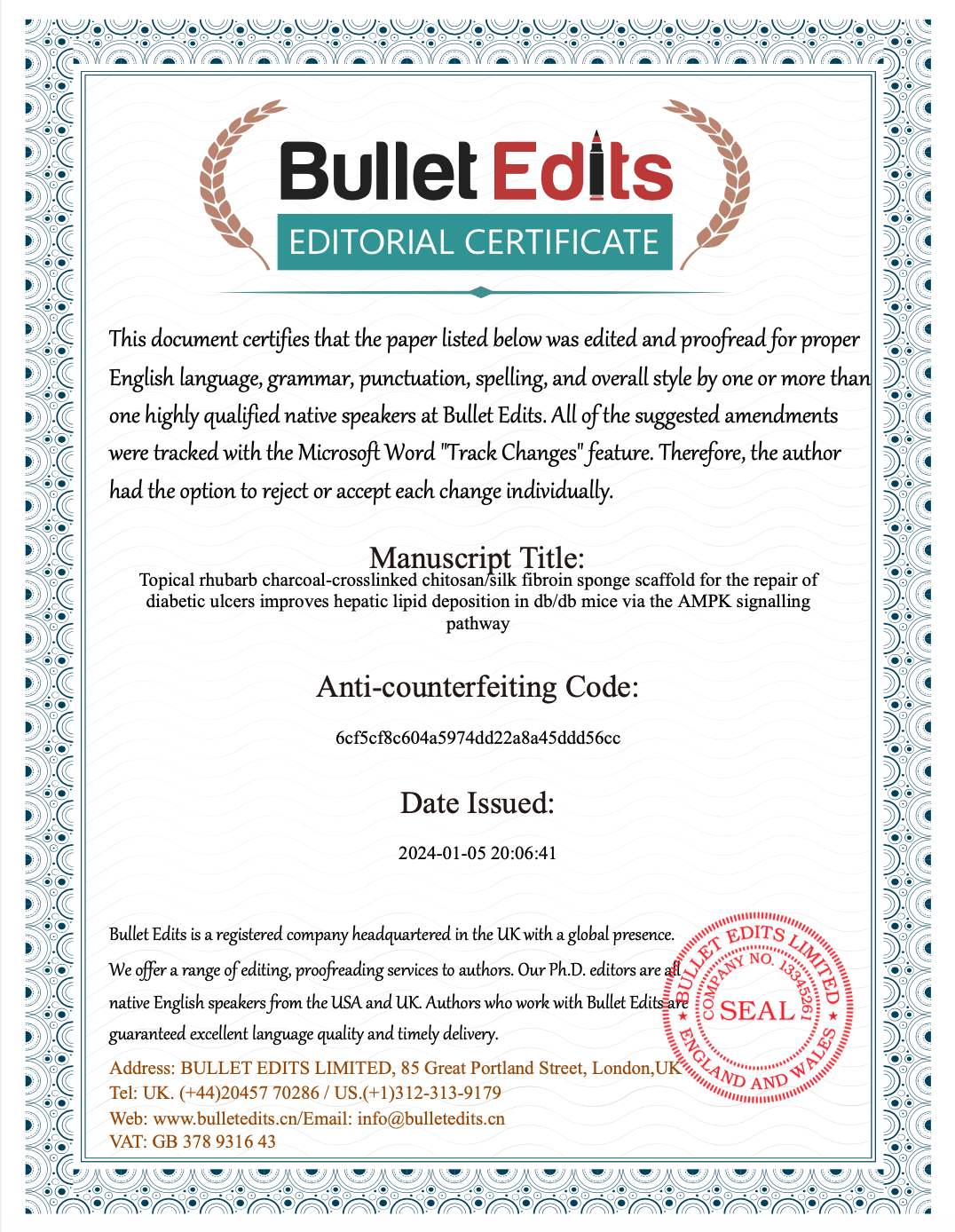


Fig S4. A certificate of language editing.


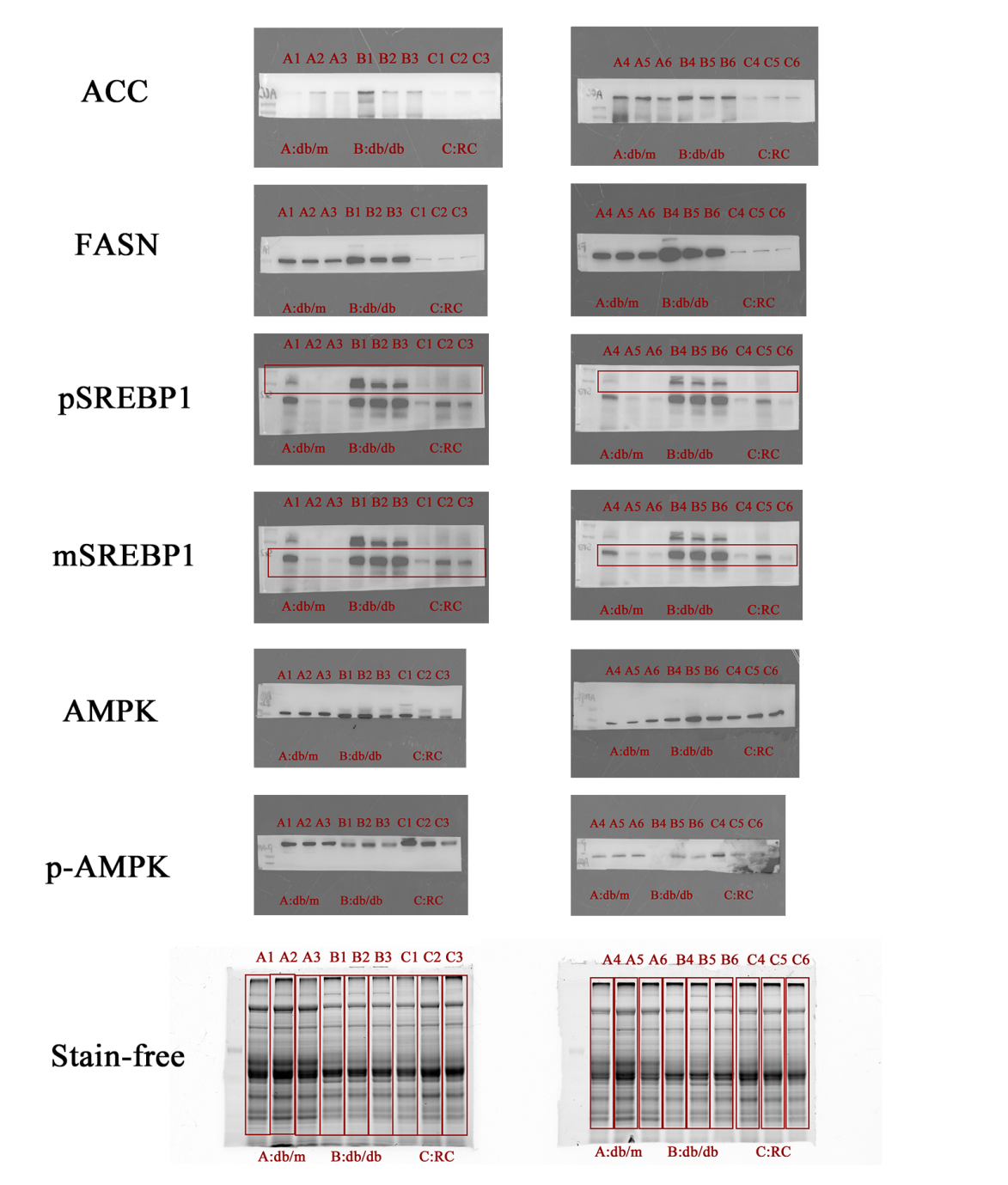


Fig S5. All the raw data for uncorrupted western blotting.
