# Supplementary material for: Topical rhubarb charcoal-crosslinked chitosan/silk fibroin sponge scaffold for the repair of diabetic ulcers improves hepatic lipid deposition in db/db mice via the AMPK signalling pathway
Source: Lipids Health Dis. 2024 Feb 20;23:52. doi: 10.1186/s12944-024-02041-z (PMC10877747; doi:10.1186/s12944-024-02041-z)
Supplement: Supplementary file 2 — Additional file 2. [file 12944_2024_2041_MOESM2_ESM.pdf]

20240201111511950390892367081472

WORD COUNT 5898

TIME SUBMITTED 01-FEB-2024 11:21AM

PAPER ID 106584133

The AMPK signalling pathway is enhanced by the topical application of a scaffold made from rhubarb charcoal-crosslinked chitosan/silk fibroin sponge for the treatment of diabetic ulcers, leading to improved hepatic lipid deposition in db/db mice.

Abstract

Background

Type 2 diabetes mellitus (T2DM) is closely linked to metabolic syndrome, characterised by insulin resistance, hyperglycaemia, abnormal lipid metabolism, and chronic inflammation. Diabetic ulcers (DUs) comprise consequential complications that arise as a result of T2DM. To investigate, db/db mice were used for the disease model. The findings demonstrated that a scaffold made from a combination of rhubarb charcoal-crosslinked chitosan and silk fibroin, designated as RCS/SF, was able to improve the healing process of diabetic wounds in db/db mice. However, previous studies have primarily concentrated on investigating the impacts of the RSC/SF scaffold on wound healing only, while its influence on the entire body has not been fully elucidated.

Material and methods

The silk fibroin/chitosan sponge scaffold containing rhubarb charcoal was fabricated in the present study using a freeze-drying approach.Subsequently, an incision with a diameter of 8 mm was made on the dorsal skin of the mice, and the RCS/SF scaffold was applied directly to the wound for 14 days.Following that, the effect of RCS/SF scaffold treatment on liver lipid metabolism was evaluated by examining serum and liver biochemistry, histopathology, qRT-PCR, immunohistochemistry, and

Western blotting.

Results

The application of the RCS/SF framework resulted in an improvement in the factors related to serum glucolipid metabolism in db/db mice.An assessment of hepatic histopathology further confirmed this enhancement.Additionally, the qRT-PCR analysis revealed that treatment with RCS/SF scaffold resulted in the downregulation of genes associated with fatty acid synthesis, fatty acid uptake, triglyceride (TG) synthesis, gluconeogenesis, and inflammatory factors.Furthermore, the positive impact of the RCS/SF framework on oxidative stress was demonstrated through the evaluation of antioxidant enzymes and lipid peroxidation.Furthermore, the network pharmacological analysis confirmed that the AMPK signaling pathway played a crucial role in alleviating NAFLD through the use of R. officinale.Support for this finding was obtained through the measurement of gene and protein expression of AMPK, sterol regulatory element binding protein 1 (SREBP1), fatty acid synthase (FASN), and acetyl CoA carboxylase (ACC).Moreover, the molecular docking studies demonstrated a strong attraction between the bioactive elements of rhubarb and the subsequent objectives of AMPK (SREBP1 and FASN).

Conclusion

By regulating the AMPK signalling pathway, the RCS/SF scaffold applied topically effectively mitigated hepatic lipid accumulation, decreased inflammation, and attenuated oxidative stress. The present study, therefore, emphasises the crucial role of the topical RCS/SF scaffold in regulating hepatic lipid metabolism, thereby confirming the concept of "external and internal reshaping".

Keywords

Rhubarb charcoal; Type 2 diabetes; AMPK signalling pathway; Hepatic lipid deposition

Introduction

Non-alcoholic fatty liver disease (NAFLD) is the most common chronic liver condition worldwide, affecting around 25% of adults (1).Conditions such as T2DM and obesity have a strong connection to NAFLD, a form of metabolic disorder.NAFLD is defined by the presence of hepatic steatosis, insulin resistance, oxidative stress, and ongoing inflammation (2, 3).The pathological development of NAFLD starts with the accumulation of fat in the liver tissue (hepatic steatosis) and can advance through different phases like non-alcoholic steatohepatitis (NASH), fibrosis, cirrhosis, and, in severe cases, hepatocellular carcinoma (4).The well-established theories of disease causation, referred to as the 'two-hit model' and 'multiple parallel hits', highlight the interconnected relationship between liver steatosis and various factors including oxidative stress, inflammation, endoplasmic reticulum stress, and dysfunction of intestinal microbes, among others (5).However, NAFLD still lacks approved treatments due to the complex nature of its progression and the presence of various associated co-morbidities (6).Hence, it is imperative to identify effective prophylactic drugs for NAFLD.

Diabetic foot ulcers (DUs), which are a complication of T2DM, have a high incidence (34% of diabetic patients globally) and present a serious risk to human health (7). Although current approaches such as debridement, infection control, maintaining a moist wound environment, and decompression are used to treat these ulcers, there are still challenges in achieving optimal efficacy and managing high costs (8, 9). Consequently, patients often experience negative financial consequences. Traditional Chinese medicine (TCM) has accumulated extensive practical knowledge over thousands of years, resulting

in a well-established theoretical framework for external treatments. Several studies have illustrated the positive impacts of TCM and the use of compound external applications in the treatment of DUs (10-13). These therapies can induce angiogenesis, promote cellular proliferation, and inhibit local inflammatory reactions, thus effectively achieving the objective of managing DUs (14).

TCM and its bioactive components have garnered significant attention in recent years due to their versatility and affordability. Owing to these attributes, TCM-based components have been loaded into hydrogels for the treatment of DUs. Numerous studies have demonstrated that hydrogel composites containing diverse TCM ingredients have potent antioxidant properties and effectively enhance the healing process of diabetic wounds (15, 16). *Rhubarb officinale* Baill refers to a group of perennial plants belonging to the genus *Rheum L.* within the Polygonaceae family. For thousands of years, rhubarb charcoal, derived from the charring process of rhubarb, has been used in China to treat burns and ulcers. In a previous study, our research group combined rhubarb charcoal with a chitosan/silk fibroin sponge scaffold and a hydrogel, using a novel TCM-based approach (17). The resulting RCS/SF scaffold was demonstrated to be highly effective in facilitating the healing of diabetic wounds.

The concept of "externally curing internal disease" was first introduced by Wu Shangxian, a physician from the Qing Dynasty.He discovered that although therapy is administered to the body's surface, it can have extensive therapeutic effects on the entire system.According to the principles TCM, a key requirement for achieving the "in vitro drug administration and systemic effect" is the drug's ability to penetrate the skin and directly reach internal organs (18, 19).In this context, previous studies not only validated the effectiveness of the topical RCS/SF scaffold in promoting wound healing in diabetic individuals, but also revealed surprising reductions in serum TG and blood glucose levels in diabetic mice.The liver, a crucial metabolic organ, plays a central role in maintaining the body's metabolic balance and regulating diverse substances (20).Furthermore, there is a notable connection between NAFLD and T2DM, both of which are linked to disruptions in hepatic glucolipid metabolism (21).Consequently, the notion of treating internal ailments from the outside was broadened to include both external and internal transformation, placing particular importance on the regulation of glucose and lipid metabolism in the liver.

The present investigation seeks to analyze the impact of applying the RCS/SF scaffold topically to improve NAFLD in db/db mice.Specifically, it improved inflammation, oxidative stress, and hepatic lipid accumulation in diabetic mice by activating the AMPK signalling pathway.This study provides the first evidence that the RCS/SF scaffold can increase hepatic lipid accumulation in db/db mice, establishing a new experimental basis for "external and internal reshaping" and presenting a novel method for enhancing disorders of hepatic lipid metabolism.

2. Materials and methods

2.1 Preparation of RCS/SF scaffold

Carboxymethyl chitosan (EFL-CMCS-200K, molecular weight = 100–200 KDa, with 90% deacetylation, and 80% carboxymethyl substitution) and water-soluble silk fibroin (EFL-SF-001, molecular weight = 6–10 KDa and 99% purity) were obtained from Suzhou Engineering for Life Technology Co. Ltd. in China.The rhubarb charcoal, sourced from the genuine medicinal plant Rheum officinale Bail in Chongqing, was produced by carbonization through stir-frying at a temperature of 200°C.Subsequently, the silk fibroin/chitosan sponge scaffolds were prepared using a freeze-drying reported in the earlier study (17).In the same way, scaffolds that carry drugs were formed by utilizing a dual physical cross-linking process to obtain the RCS/SF scaffold.To the CS/SF mixture, 100 mg of rhubarb charcoal was added as part of this procedure.

2.2 Animals

We acquired twelve male db/db mice and six male db/m mice, both at the age of 8 weeks, from Jiangsu Huachuang Xinnuo Pharmaceutical Technology Limited Company located in Jiangsu, China.In the animal facility of Chongqing Medical University, the mice were accommodated in Specific Pathogen-Free facilities.Every individual mouse was housed in a separate enclosure, maintaining a temperature of 22 ± 2°C, and ensuring a 12-hour cycle of light and darkness.

2.3 Experimental groups

Following a week of acclimatization, male db/db mice were randomly divided into two groups: the model group (db/db, n = 6) and the administration group (RC, n = 6).The experimental group was comprised of db/m mice (db/m, n = 6).Under isoflurane anaesthesia, a puncture biopsy instrument was used to create an 8-mm diameter wound on the back of each mouse with moderate force (22).Following that, group RC received RCS/SF scaffold, whereas the remaining two groups were administered normal saline solution at the site of wound.The treatment lasted 14 days, with food and water intake recorded every three days.For precise water intake measurement over a three-day span, water bottles were directly weighed, and intake was calculated by comparing weights before and after the period."All animal tests adhered to the ethical principles for animal research and received approval from The Ethics Committee of Chongqing Medical University (Approval No."2022168).

2.4 Serum biochemical analysis

The biochemical assay kits were utilized following the guidelines provided in the kit manual from Nanjing Jiancheng Bioengineering Institute located in Nanjing, China.Various tests were employed to assess the levels of total cholesterol (TC), low-density lipoprotein cholesterol (LDL-C), high-density lipoprotein cholesterol (HDL-C), TG, and glucose in the serum.Measurements were collected for TC (A111-1-1), LDL-C (A113-1-1), HDL-C (A112-1-1), TG (A110-1-1), and glucose (A154-1-1) through the process of taking measurements.The absorbance values of TC, TG, and glucose were measured at wavelengths of 505 nm, 510 nm, and 505 nm, respectively, using a versatile microplate reader.In addition, the LDL-C and HDL-C measurements were carried out at a wavelength of 546 nm.The kits used were obtained solely from Nanjing Jiancheng Bioengineering Institute located in Nanjing, China.

2.5 Liver biochemical analysis

Nanjing Jiancheng Bioengineering Institute offered the aspartate aminotransferase (AST) (C010-2-1), alanine aminotransferase (ALT) (C009-2-1), and TG (A110-1-1).The test liquid was prepared following the instructions given in the kit with strict adherence.To summarize, approximately 30 milligrams of liver tissue were assessed and subsequently combined with a volume of phosphate buffer saline (0.1 mol/L pH 7.4) that was nine times greater, while maintaining a ratio of 1 gram to 9 milliliters.Grinding beads were then added to ensure complete homogenization of the tissues.The blend was positioned on the Four-Dimensional Rotating Mixer (Beyotime, Shanghai, China) and allowed to incubate for the duration of the night at a temperature of 4°C.After the liver tissues were homogenized and centrifuged, the resulting supernatant was collected.In order to standardize, the protein levels in liver tissue were measured using a BCA Protein Concentration Assay kit (#P0010, Beyotime, Shanghai, China).Obtaining the absorbance measurements for ALT, AST, and TG involved utilizing a multi-function microplate reader configured to operate at a wavelength of 510 nm.

2.6 Measurement of antioxidant enzymes and lipid peroxidation

Approximately 30 mg of liver tissue homogenate was measured, and the liquid portion was obtained after centrifugation.Subsequently, the hepatic concentrations of Catalase (CAT), Malondialdehyde (MDA), Total Superoxide Dismutase (SOD), and NAD+/NADH were assessed by employing CAT, MDA(A007-1-1, A003-1-2, Nanjing, China), SOD, and NAD+/NADH assay kits (S0101S, S0175, Beyotime Biotechnology, Shanghai, China) according to the provided guidelines.

2.7 Hepatic histopathological analysis

The mouse liver tissue was fixed using a 4 paraformaldehyde solution.Subsequently, it underwent a drying process, was enveloped in paraffin, and subsequently divided into segments.Afterwards, the samples were cut into 4 µm slices using a manual rotary microtome (Leica RM2235, Wetzlar, Germany) and subsequently treated with haematoxylin and eosin (H&E) stain.The staining procedure yielded a blue colouration in the nucleus and a red colouration in the cytoplasm.Furthermore, the combined occurrence of steatosis, ballooning, and lobular inflammation was evaluated utilizing the NAFLD activity score (NAS) algorithm (23).Conversely, the liver glycogen content was assessed by employing periodic acid-Schiff (PAS) staining.After being moisturized, the paraffin slices underwent treatment with periodic acid and Schiff's reagent for 5 minutes and 15 minutes, respectively.They were then stained with haematoxylin for 2 min.The glycogen exhibited a purplish-red hue, whereas the nucleus was stained with a blue colour.The liver tissue was initially preserved in a 4 paraformaldehyde solution.It was then subjected to a process of gradual removal of water using a sucrose solution ranging from 30% to 10%.Finally, the tissue was embedded using

TissueTek OCT Compound.The tissue was subsequently divided into 6μm slices using a cryotome (Leica CM1860, Wetzlar, German) and then treated with Oil Red O staining.The staining with Oil Red O was performed according to the previously established protocol (24).The Oil Red O working solution was prepared by dissolving 1g of Oil Red O powder in 100 mL of isopropanol, which was then combined with distilled water in a 3:2 ratio.Following the filtration process, the solution was subjected to staining for a duration of 10 min.The process of differentiation was performed using a solution containing 60% isopropanol, while the nuclei were stained with haematoxylin as a counterstain.The findings revealed the presence of red lipids and blue nuclei.Moreover, the liver was examined for pathological changes using a light microscope from Olympus BX51 in Tokyo, Japan. The observations were recorded at both 200X and 400X levels of magnification.The liver tissue was additionally examined for the accumulation of glycogen and lipid droplet using the software called National Institutes of Health Image J (NIH, USA).The analysis results were quantified as the proportion of glycogen and lipid droplets that were present in the liver tissue relative to the total area of the visual field.

2.8 Immunohistochemistry

Paraffin slices of liver tissues measuring 4μm were subjected to a meticulous process, which involved de-paraffinisation, hydration, and a 10-min treatment with endogenous peroxidase blockers.Afterward, the slices underwent antigen restoration for a duration of 5 minutes.Following a 30-minute period with bovine serum albumin (BSA), the slices were left to incubate overnight at 4°C with rabbit anti-TNFα antibody (GB11188-100, Servicebio, Wuhan, China, 1 300).Afterward, the liver sections were exposed to secondary antibodies that were linked with horseradish peroxidase for a period of 30 minutes at room temperature.Following this, the sections were stained using a DAB working solution (AFIHC004, AiFang biological, Changsha, China) and subsequently counterstained with haematoxylin.In addition, the segments were examined using a light microscope (Olympus BX51, Tokyo, Japan) and images were taken at both 100X and 400X amplification.Subsequently, the images were analyzed and measured using the ImageJ software (NIH, USA).

Quantitative PCR performed in real-time with a value of 2.9

The liver was subjected to total RNA extraction using AG RNAex Pro Reagent (AG21102, Accurate Biotechnology, Changsha, China).The concentration of RNA was measured using the NanoDrop 2000 spectrophotometer and standardized across all samples.Subsequently, the Evo M-MLV reverse transcription Premix kit (AG11705, Accurate Biotechnology, Changsha, China) was utilized to generate complementary deoxyribonucleic acid (cDNA) for quantitative real-time PCR.Quantitative RT-PCR reactions were prepared using the premixed qPCR kit (AG11740, Accurate Biotechnology, Changsha, China) containing SYBR Green Pro Taq HS.To amplify these reactions, the CFX-96 real-time PCR system

from BioRad in Hercules, CA, USA was utilized.The measurement of mRNA levels was conducted utilizing the 2-ΔΔCt technique and standardized against the ribosomal protein lateral stalk subunit P0 (Rplp0), which functioned as a reference gene.The primer sequences utilised in the experiment are presented in Table S1.

2.10 Western blotting

Samples of liver weighing 30 mg were homogenized with RIPA buffer (Sangon Biotech, Shanghai, China) containing protease and phosphatase inhibitors.The protein samples were homogenised for 3 min using a TissueLyser.Subsequently, they were centrifuged at a 12,000 rpm and a temperature of 4[illegible] for 10 min (#5810, Eppendorf, Hamburg, Germany).The total protein was quantified using the BCA protein concentration determination kit (#P0010, Beyotime, Shanghai, China) after obtaining the resulting supernatants.Afterwards, the specimens underwent separation through the utilization of 10-12% SDS-PAGE and TGX Stain-free gels (1610183, BioRad, Hercules, CA, USA), and subsequently moved onto PVDF membranes (Millipore, Bedford, MA, USA).The membranes were blocked in a Tris Buffered Saline with Tween (TBST) solution, containing 5% non-fat dry milk (or 5% BSA for the phosphor-AMPK), for a period of 1 hour.Subsequently, the specimens were allowed to incubate overnight at a temperature of 4°C using a primary antibody.The blot was then subjected to treatment with horseradish peroxidase-conjugated anti-IgG.Detection was carried out using ECL (BL520A, Biocharp, Beijing, China).Moreover, the stain-free technology (BioRad) was employed for normalizing total protein concentration (25).The ChemiDoc Imaging System (BioRad, Hercules, CA, USA) was used for image acquisition, while the ImageJ software (NIH, USA) was employed to quantitatively analyze the intensities of the target bands.The antibodies used in this study were AMPKα (AF6423, Affinity, Changzhou, China, 1:1000), Phospho-AMPKαThr172 (#AF3423, Affinity, USA, 1:1000), SREBP1 (sc-13551, Santa Cruz, CA, USA, 1:500), FASN (10624-2-AP, Proteintech, Wuhan, China, 1:5000), and ACC (3676s, Cell Signaling Technology, Danvers, MA, USA, 1:1000).

2.11 Network pharmacology analysis

To explore the various components, crucial targets, and possible mechanisms of rhubarb in fighting NAFLD, the network pharmacology method was employed."Rhubarb" and "non-alcoholic fatty liver disease" served as search keywords in relevant databases.The active components of rhubarb were acquired from the Traditional Chinese Medicine Database and Analysis Platform (TCMSP, https //tcmsp-e.com/) using a drug-likeness (DL) threshold of ≥0.18 as a criterion.To screen these substances and their potential targets, the Uniprot database (https://www.uniprot.org/) was employed.At the same time, NAFLD targets were acquired by searching the OMIM (https //www.omim.org/) and GeneCards (https //www.genecards.org/) databases.Subsequently, by

creating a Venn diagram, the shared targets between rhubarb and NAFLD were identified.Moreover, the primary objectives were acquired from the STRING (https //string-db.org/) database.To evaluate the potential targets, the analyses included the utilization of Gene ontology (GO) enrichment and Kyoto Encyclopaedia of Genes and Genome (KEGG) pathway assessments.

2.12 Molecular docking analysis

AutoDock Vina (1.1.2), a molecular docking software, was utilized to verify the interaction activities between the active components and targets.The mol2 format compounds were acquired from the official TCMSP website, while the primary target proteins were obtained from the PDB database at http //www.rcsb.org/.The proteins were brought into AutoDocktools (v1.5.6) and saved in the format 'pdbqt'.The interaction patterns were then analysed using PyMOL 2.3.0.

2.13 Statistics analysis

GraphPad Prism 9.0 software (GraphPad Software Inc.) was utilized for the statistical analysis.The means ± standard errors of the mean (SEM) were used to display all graphical data.Comparisons between groups were examined using a one-way ANOVA, followed by Dunnett's test.Consequently, the values that had a significance level below $P < 0.05$ were deemed statistically significant.

3. Results

The use of RCS/SF scaffold leads to a reduction in both the weight and index of the liver in db/db mice.

The diabetic mice displayed noticeably increased body weight, food intake, and water intake compared to the db/m mice over a span of 14 days (Figs.1A-C).However, the administration of RCS/SF scaffold did not have any noticeable effect on the overall condition of the db/db mice (Figs.1A-C).In addition, the graphs illustrating the average consumption of food and water showed no discernible difference between the db/db group and the RC group (Figs.1D, E), suggesting that diabetic mice had similar energy intake.Moreover, the hepatic morphological photographs clearly demonstrated that the db/m group displayed a sleek and supple texture.In contrast, the db/db group displayed a noticeably bigger dimension, distinguished by a surface embellished with prominent white, glossy, grainy projections.The application of RCS/SF scaffold treatment effectively reduced the prominent roughness on the liver surface of diabetic mice, while also reducing the enlarged size of the liver (Fig.1F).Furthermore, the validity of this outcome was confirmed through the notable decrease in liver weight and liver index following the intervention in the RC group.(Figs.1G, H).The collective

findings suggest that the utilization of RCS/SF scaffold during treatment resulted in a decrease in both liver mass and liver ratio, while having no effect on the overall body mass.

The RCS/SF scaffold enhances glucolipid metabolism disorders in db/db mice.

The RCS/SF scaffold demonstrated a significant hypoglycaemic effect on diabetic mice.As depicted in Fig.2A, the serum glucose levels in the RC group were significantly lower compared to the db/db group.There was no notable disparity in serum TC between the RC group and the db/db group regarding their influence on blood lipids (Fig.2C).However, the RC group experienced significant reductions in serum TG and serum LDL-C levels (Figs.2B, D).Moreover, the RCS/SF scaffold intervention effectively reversed the reduced HDL-C levels in the db/db cohort (Fig.2E).Importantly, a notable decrease in serum ALT and AST levels was observed in diabetic mice receiving treatment with RCS/SF scaffold (Figs.2F, G).To summarise, these findings indicate that the RCS/SF scaffold effectively mitigates glycolipid abnormalities in diabetic mice.

3.3 RCS/SF scaffold ameliorates liver injury and lipid deposition in db/db mice

A histopathological examination of the liver was conducted to study the effects of the RCS/SF scaffold in reducing liver damage and lipid buildup in db/db mice.NAFLD encompasses a range of conditions that involve the buildup of fat in the liver (steatosis), inflammation causing cell death (necrotizing inflammation), and mild inflammation resulting in hepatocellular injury (3).H&E staining revealed notable steatosis and ballooning in the liver of db/db mice.However, the administration of RCS/SF scaffold mitigated this pathological condition (Figs.3A, D).The db/db cohort exhibited a notable augmentation and circularity of hepatic cells, accompanied by a substantial rise in lipid droplets, as evidenced by the staining using oil red O.However, the introduction of RCS/SF framework resulted in a notable reduction in both the size and quantity of lipid droplets (Figs.3B, E).Furthermore, the PAS staining exhibited a plentiful presence of purplish-red glycogen particles in the liver tissue of db/m mice, while a significant reduction was noted in db/db mice.Nonetheless, the application of the RCS/SF framework effectively restored the reduced glycogen storage (Figs.3C, F).In the meantime, the liver biochemistry test revealed a notable decrease in hepatic TG levels in db/db mice that received the RCS/SF scaffold treatment (Fig.3G).In addition, the liver of db/db mice exhibited significantly increased levels of ALT and AST, which are widely acknowledged as indicators of liver injury, when compared to db/m mice.Nevertheless, the administration of RCS/SF scaffold treatment effectively inhibited the increase in ALT and AST level (Figs.3H, I).In general, the data indicates that the RCS/SF framework improves the accumulation of fats in the liver and decreases liver injury in db/db mice.

3.4 RCS/SF scaffold mitigates hepatic oxidative stress in db/db mice

To evaluate the impact of the RCS/SF framework on liver oxidative stress in db/db mice, a research was carried out, taking into account the significant role of oxidative stress in the advancement of NAFLD (26).As depicted in Fig.In contrast to db/m mice, db/db mice exhibited a significant decrease in the liver levels of SOD, CAT, and NAD+/NADH.However, the administration of RCS/SF scaffold resulted in elevated levels of these antioxidant enzymes in the liver tissue (Figs.4A-C).Moreover, the results also demonstrated a significant reduction in elevated MDA levels in db/db mice following treatment with the RCS/SF scaffold (Fig.4D).Hence, the aforementioned data suggests that the RCS/SF scaffold has a protective effect on NAFLD by controlling the liver's ability to counteract oxidative stress in db/db mice.

The RCS/SF scaffold influences the expression of genes associated with glycolipid metabolism in db/db mice.

For the purpose of investigation, gene expression levels were measured using qRT-PCR to assess the effect of the RCS/SF scaffold in reducing hepatic lipid accumulation in db/db mice.The findings indicate a notable decrease in the mRNA levels of nuclear receptor subfamily 1 group h member 3 (Nr1h3), ATP citrate lyase (Acly), and pyruvate kinase liver and red blood cell (Lpk, Pklr) genes, which are controlled by MLX interacting protein-like (ChRebp, Mlxipl), in db/db mice that received RCS/SF scaffold treatment (Fig.5A).Moreover, the RCS/SF scaffold treatment successfully reduced the increased mRNA levels of genes in db/db mice associated with the transport of fatty acids, specifically the CD36 molecule (Cd36), solute carrier family 27 member 4 (Fatp4, Slc27a4), and solute carrier family 27 member 5 (Fatp5, Slc27a5) (Fig.5B).The expression of genes implicated in glucose metabolism was also assessed.Although it did not cause a change in the expression of solute carrier family 2-member 2 (Glut2, Slc2a2) in db/db mice, the RCS/SF scaffold significantly reduced the mRNA expression of phosphoenolpyruvate carboxykinase 1 (Pepck, Pck1) (Fig.5C).In addition, the levels of diacylglycerol O-acyltransferase 2 (Dgat2) and mannoside acetylglucosaminyltransferase 2 (Mgat2), essential enzymes in TG synthesis, were examined.A notable decrease in the expression of these enzymes was observed when utilizing the RCS/SF scaffold.In db/db mice, the RCS/SF scaffold affects the function of genes associated with glycolipid metabolism, resulting in the suppression of fatty acid synthesis, fatty acid transportation, TG synthesis, and gluconeogenesis inhibition.

3.6 RCS/SF scaffold ameliorates hepatic inflammation in db/db mice

To investigate the impact of the RCS/SF scaffold on improving liver function, a study was conducted to assess its influence on liver inflammation.The qRT-PCR findings in db/db mice showed a significant increase in the mRNA relative expression of inflammatory markers, such as tumor necrosis factor-alpha (Tnfα), interleukin 6 (Il-6), interleukin 10 (Il-10), and interleukin 1 beta (Il-1β).However, the application of RCS/SF scaffold led to a notable decrease in the levels of Tnfα and

Il-1β expression (Fig.6A).Moreover, analysis of liver tissue using immunohistochemistry revealed a significant rise in Tnfα protein expression in the db/db group in comparison to the db/m group.Fig. shows a significant decrease in the expression of RCS/SF scaffold following intervention, compared to the db/db group.6B).As observed, the findings suggest that the RCS/SF scaffold has a positive impact in improving hepatic inflammation.

3.7 RCS/SF scaffold activates AMPK/SREBP1 pathway in db/db mice

To elucidate the molecular mechanisms underlying RCS/SF scaffold's action against NAFLD, network pharmacology was employed for prediction (Fig.S1).KEGG Pathway analysis revealed 129 pathways associated with rhubarb's treatment of NAFLD (Fig.S2).Fig. displays the top 20 pathways.7B.The vital role of AMPK signalling in regulating the progression of NAFLD is widely recognized (27).Therefore, subsequent molecular experiments were performed to affirm this connection.The qRT-PCR analysis indicated a notable rise in the relative mRNA expression of Srebf1, Acaca, and Fasn in the livers of db/db mice, which was effectively inhibited by the RCS/SF scaffold.Meanwhile, there was no notable variation in the comparative mRNA expression of prkaa1 among the three groups (Fig.7C).Correspondingly, the RCS/SF scaffold significantly inhibited the levels of SREBP1, FASN, and ACC proteins, while enhancing the expression of p-AMPK (Fig.7D, E).In summary, the findings indicate that the RCS/SF scaffold exerts alleviating hepatic lipid deposition effects by modulating the AMPK/SREBP1 pathway.

3.8 Molecular docking verification

Based on the aforementioned analysis, the top five important targets were selected from the 22 active ingredients screened for rhubarb. These targets were chosen based on their higher degree values and will undergo semi-flexible docking. The details can be found in Tables S2 and S3. Affinity is employed to indicate the efficacy of a small molecule's binding to its target protein. A negative binding energy indicates that a small molecule can readily bind to its target protein, with a lower value indicating a higher probability of

binding. The docking analysis demonstrated that emodin formed hydrogen bonds with specific amino acid residues (ILE-2068, GLY-1895, PHE-1896, LEU-1971, and GLY-2061) of the FASN protein. The calculated docking energy for this interaction was -9.5 kcal/mol (Fig. 8A). In addition, aloe-emodin established hydrogen bonds with LYS-365 and LEU-349 of SREBP1, resulting in a docking energy of -7.4 kcal/mol (Fig. 8B). These results indicate that the selected targets display a robust binding affinity with their corresponding active compounds, offering further proof of the reliability of the AMPK/SREBP1 pathway predictions made using network pharmacology, aligning with prior molecular experiments.

Discussion

Recent studies have indicated that rhubarb possesses multiple active compounds that demonstrate diverse pharmacological effects, including but not limited to anti-inflammatory, anti-cancer, antioxidant, hepatoprotective, immune-modulating, lipid-lowering, and laxative properties (28-33).Additionally, multiple animal experiments have demonstrated that rhubarb and its active components have a significant impact on lipid metabolism (34-36).Specifically, they regulate this process by inhibiting lipogenesis, increasing lipolysis, and reducing lipid deposition.In a previous study, a biocompatible CS/SF scaffold was developed by incorporating rhubarb charcoal into a chitosan hydrogel scaffold, thereby demonstrating its antibacterial and anti-inflammatory efficacy (17).The current study has shown that the RCS/SF framework has a beneficial effect on the accumulation of fats in the liver, inflammation, and oxidative stress in db/db mice.Accordingly, additional investigations were conducted in the current study to explore the potential impacts by examining the regulation of the AMPK/SREBP1 pathway, offering insights into the molecular mechanisms underlying these observed enhancements.

Db/db mice, characterised by mutations in the leptin receptor gene, exhibit a disrupted leptin

signalling pathway, resulting in obesity, insulin resistance, hyperglycaemia, lipid metabolism disorders, inflammation, and oxidative stress.As a result, these mice are often employed as NAFLD research models for NAFLD (37).The pathogenesis of NAFLD is complex and encompasses multiple factors.In addition to hepatic steatosis caused by the accumulation of hepatic triglycerides and de novo lipogenesis (DNL) (38), the liver can also accumulate toxic lipid species if it has difficulty processing primary metabolic energy substrates (39).The accumulation of these substance causes stress, damage, and death in liver cells leading to inflammation and oxidative stress (40).Ultimately, this sequence of occurrences has the potential to result in the formation of cirrhosis and hepatocellular carcinoma (41, 42).

AMPK, a serine/threonine protein kinase that is widely preserved, is essential for the regulation of cellular energy balance, maintenance of cellular energy equilibrium, and control of metabolism (43).An increase in the intracellular ratio of Adenosine Monophosphate (AMP) to Adenosine Triphosphate (ATP) indicates a decrease in cellular energy levels, which leads to the activation of AMPK (44).When activated, AMPK hinders the transcriptional function and decreases the SREBP1 expression.By inhibiting SREBP1, a key regulator of hepatic DNL, the transcriptional activities of FASN and ACC are subsequently reduced (45).As a result, AMPK is considered a highly promising therapeutic target for NAFLD (46, 47).Recent studies suggest that AMPK has the ability to rectify energy metabolism abnormalities within tumors.By orchestrating processes like the cell cycle, metabolism, and autophagy in cells, AMPK plays a pivotal role in combating both tumors and drug resistance (48-52).Prior research has confirmed that emodin, a primary active compound found in rhubarb, acts as a regulator of AMPK (53).By activating AMPK, emodin was shown in the study to cause a reduction in the levels of SREBP1 and FASN proteins in the hepatocytes of rats that were fed a high-fat diet (HFD).As expected, the study utilised network pharmacology predictions to determine that the AMPK signalling pathway is significantly involved in the therapeutic effects of rhubarb for NAFLD, as indicated by the KEGG enrichment results.Additional trials additionally confirmed that the utilization of RCS/SF framework successfully inhibited the manifestation of SREBP1, FASN, and ACC.

Oxidative stress occurs when there is an imbalance in the cellular antioxidant equilibrium due to the excessive generation of reactive oxygen species (ROS) and reactive nitrogen species (RNS) inside cells (54, 55).Consequently, vital components of cells such as fats, amino acids, and DNA are prone to damage.The build-up of fats in liver cells in NAFLD results in the generation of detrimental lipid by-products like diacylglycerol, ceramides, cholesterol, and free fatty acids (56).At the same time, conditions like insulin insensitivity, irregularities in glucose processing, and abnormal cholesterol processing collectively interfere with the regular operation of mitochondria, leading to an elevated generation of ROS and the onset of oxidative stress (57, 58).During periods

of oxidative stress, ROS directly target lipid molecules within the cell membrane, causing lipid peroxidation.This process results in the breakdown of lipid molecules and the creation of lipid peroxidation products, such as MDA.In addition, SOD converts superoxide radicals into hydrogen peroxide (H_2O_2), which is subsequently broken down by enzymes such as glutathione peroxidase (GPx) or CAT (59, 60).When the organism undergoes oxidative stress, it triggers both enzymatic and non-enzymatic mechanisms to counteract the production of ROS (61).Previous studies have confirmed that emodin has the ability to reduce hepatic oxidative stress in HFD-fed mice (62).The current study's findings are consistent with the research results, indicating a notable reduction in MDA concentrations in the livers of db/db mice after administering RCS/SF scaffold treatment, indicating a decrease in oxidative stress.Concurrently, the RCS/SF scaffold also facilitated the restoration of enzyme activity, specifically in CAT, SOD, and the NAD+/NADH ratio, suggesting a beneficial impact on antioxidant defence mechanisms and cellular energy metabolism.

Moreover, the release of inflammatory mediators exacerbates oxidative stress (63).Oxidative stress triggers lipid peroxidation, leading to the release of pro-inflammatory substances like cytokines (IL-1β, IL-6, and TNF-α) and chemokines.The liver (64) benefits from these compounds by promoting the enlistment and stimulation of immune cells.The excessive build-up of lipids in the liver is closely connected to hepatocyte injury and the activation of Kupffer cells, which in turn triggers inflammatory responses that exacerbate hepatic disease (65).Prior research has shown that emodin possesses the capacity to decrease the production of inflammatory factors in mice experiencing liver injury induced by lipopolysaccharide (LPS) (66).According to the ongoing inquiry, db/db mice exhibited a notable increase in the mRNA expression levels of inflammatory markers (Tnfα, Il-6, and Il-1β) when compared to db/m mice.Nevertheless, the RC group experienced a substantial decrease in these levels.Furthermore, the immunohistochemical findings additionally exhibited a notable reduction in the expression of Tnfα protein after the application of RCS/SF scaffold treatment when compared to the db/db group.

In addition to the above, network pharmacology was utilized in the current study to predict the specific targets affected by rhubarb during the treatment of NAFLD.The analysis of KEGG enrichment indicated a notable association between the utilization of rhubarb for NAFLD and the activation of the AMPK signalling pathway.This was further validated from additional molecular docking studies, which confirmed the binding of five bioactive compounds in rhubarb with two downstream targets of AMPK, namely SREBP1 and FASN.Accordingly, the findings demonstrated that all five active components exhibited a strong binding activity with SREBP1 and FASN, as evidenced by their negative binding energy.Hence, the utilization of these two approaches, in conjunction with molecular investigations, aids in confirming and establishing a scientific basis for understanding the mechanism of the RCS/SF framework in tackling NAFLD.

Based on the research results, the topical application of the RCS/SF scaffold effectively reduces hepatic lipid accumulation, inflammation, and oxidative stress by activating the AMPK signaling pathway. This leads to an overall enhancement in NAFLD in db/db mice, as shown in Figure.9).Furthermore, the utilization of the 'external and internal restructuring' concept clarifies the potential of the RCS/SF framework to provide a healing impact that focuses on various facets and measures.In summary, it was hypothesized that the RCS/SF framework had the ability to function as an antimicrobial bandage for diabetic wound treatment and also had a beneficial influence on reducing hepatic lipid accumulation by controlling lipid metabolism.As noted, the initial scientific findings not only back up this hypothesis but also endorse the potential utilization of the RCS/SF framework for clinical purposes.

Study strength and limitations

This study reveals the effectiveness of applying RCS/SF scaffolds in significantly reducing hepatic lipid deposition in db/db mice for the first time.The mechanism appears to be closely associated with the regulation of the AMPK signaling pathway.The research also preliminary explores the scaffold's impact on inflammation, oxidative stress, and the regulation of genes related to glycolipid metabolism, aligning with the "external and internal reshaping" theory.However, the study has several limitations, and more in-depth research is needed to explain the specific mechanisms involved.Additionally, exploring the impact of RCS/SF scaffold on skeletal muscle or other metabolism-related organs is worthy of further investigation.

Conclusions

To summarize, this research clarifies that the RCS/SF scaffold, when applied topically, successfully reduces inflammation, oxidative stress, and accumulation of fat in the liver of db/db mice by regulating the AMPK signaling pathway.This lays the groundwork for its potential therapeutic application in managing metabolic disorders associated with diabetic wound healing.Additionally, preceding investigations have underscored the superior wound healing properties of RCS/SF scaffold.Consequently, this research establishes a robust theoretical framework for the clinical implementation of RCS/SF scaffold as an innovative dressing for diabetic wounds complicated by metabolic issues.

20240201111511950390892367081472

ORIGINALITY REPORT

18%
SIMILARITY INDEX

PRIMARY SOURCES

1	Shang Wang, Yi Zhang, Yan Shi, Qifeng He, Qi Tan, Ze Peng, Yuzhe Liu, Dong Li, Xuezhi Li, Dazhi Ke, Jianwei Wang. "Rhubarb charcoal-crosslinked chitosan/silk fibroin sponge scaffold with efficient hemostasis, inflammation, and angiogenesis for promoting diabetic wound healing", International Journal of Biological Macromolecules, 2023<br>Crossref	70 words — 1%
2	www.frontiersin.org<br>Internet	66 words — 1%
3	www.mdpi.com<br>Internet	56 words — 1%
4	www.dovepress.com<br>Internet	46 words — 1%
5	mdpi-res.com<br>Internet	44 words — 1%
6	worldwidescience.org<br>Internet	43 words — 1%
7	link.springer.com<br>Internet	31 words — < 1%
8	Lee, Young A, Eun Ju Cho, and Takako Yokozawa. "Effects of Proanthocyanidin Preparations on	30 words — < 1%

Hyperlipidemia and Other Biomarkers in Mouse Model of Type 2 Diabetes", Journal of Agricultural and Food Chemistry, 2008.
Crossref

9 Yu, Fei, Bao-ying Li, Xiao-li Li, Qian Cai, Zhen Zhang, Mei Cheng, Mei Yin, Jun-fu Wang, Jian-hua Zhang, Wei-da Lu, Rui-hai Zhou, and Hai-qing Gao. "Proteomic Analysis of Aorta and Protective Effects of Grape Seed Procyanidin B2 in db/db Mice Reveal a Critical Role of Milk Fat Globule Epidermal Growth Factor-8 in Diabetic Arterial Damage", PLoS ONE, 2012.
Crossref
30 words — < 1%

10 e-century.us
Internet
29 words — < 1%

11 Yuejin Ji, Yajun Liu, Jingyi Hu, Cheng Cheng, Jing Xing, Lei Zhu, Hong Shen. "Exploring the Molecular Mechanism of Astragali Radix-Curcumae Rhizoma against Gastric Intraepithelial Neoplasia by Network Pharmacology and Molecular Docking", Evidence-Based Complementary and Alternative Medicine, 2021
Crossref
27 words — < 1%

12 www.science.gov
Internet
27 words — < 1%

13 "Minutes of the 44th Genral Assembly of the European Association for the Study of Diabetes", Diabetologia, 2009
Crossref
26 words — < 1%

14 Longxin Qiu, Jianhui Lin, Fangui Xu, Yuehong Gao, Cuilin Zhang, Ying Liu, Yu Luo, James Y. Yang. " Inhibition of Aldose Reductase Activates Hepatic Peroxisome Proliferator-Activated Receptor- and Ameliorates
26 words — < 1%

Hepatosteatosis in Diabetic db/db Mice ", Experimental Diabetes Research, 2012
Crossref

---

15 www.researchgate.net 25 words — < 1%
Internet

---

16 topsecretapiaccess.dovepress.com 24 words — < 1%
Internet

---

17 Ying Liu, Mingming Zhai. "Pulsed Electromagnetic Fields Alleviates Hepatic Oxidative Stress and Lipids Accumulation in db/db mice", Cold Spring Harbor Laboratory, 2020 23 words — < 1%
Crossref Posted Content

---

18 Aili Cao, Li Wang, Xia Chen, Hengjiang Guo, Shuang Chu, Xuemei Zhang, Wen Peng. "Ursodeoxycholic Acid Ameliorated Diabetic Nephropathy by Attenuating Hyperglycemia-Mediated Oxidative Stress", Biological & Pharmaceutical Bulletin, 2016 22 words — < 1%
Crossref

---

19 ebin.pub 22 words — < 1%
Internet

---

20 5dok.org 20 words — < 1%
Internet

---

21 assets.researchsquare.com 20 words — < 1%
Internet

---

22 www.researchsquare.com 19 words — < 1%
Internet

---

23 www.jfda-online.com 17 words — < 1%
Internet

24	Fengjuan Tang, Yarong Hao, Xue Zhang, Jian Qin. "Effect of echinacoside on kidney fibrosis by inhibition of TGF-β1/Smads signaling pathway in the db/db mice model of diabetic nephropathy", Drug Design, Development and Therapy, 2017<br>Crossref	16 words — < 1%
25	www.nature.com<br>Internet	16 words — < 1%
26	Ana Chocarro-Calvo, Miguel Jociles-Ortega, Jose Manuel Garcia-Martinez, Pakavarin Louphrasitthiphol et al. "Phenotype-specific melanoma uptake of fatty acid from human adipocytes activates AXL and CAV1-dependent beta-catenin nuclear accumulation", Cold Spring Harbor Laboratory, 2024<br>Crossref Posted Content	15 words — < 1%
27	Min Yan, Huan Qi, Jia Li, Guozhu Ye, Yaping Shao, Tongming Li, Jing Liu, Hai-long Piao, Guowang Xu. "Identification of <i>SPOP</i> related metabolic pathways in prostate cancer", Oncotarget, 2017<br>Crossref	15 words — < 1%
28	Minfeng Zhou, Jinxiao Li, Dan Luo, Haiming Zhang, Zhaomin Yu, Youlin Chen, Qiumeng Li, Fengxia Liang, Rui Chen. "Network Pharmacology and Molecular Docking–Based Investigation: Prunus mume Against Colorectal Cancer via Silencing RelA Expression", Frontiers in Pharmacology, 2021<br>Crossref	15 words — < 1%
29	Chen Liang, Yan Li, Miao Bai, Yanxin Huang et al. "Hypericin attenuates nonalcoholic fatty liver disease and abnormal lipid metabolism via the PKA-mediated AMPK signaling pathway in vitro and in vivo", Pharmacological Research, 2020	14 words — < 1%

Crossref

30	synapse.koreamed.org<br>Internet	14 words — < 1%
31	biosignaling.biomedcentral.com<br>Internet	13 words — < 1%
32	oxfordjournals.org<br>Internet	13 words — < 1%
33	www.biorxiv.org<br>Internet	13 words — < 1%
34	Da Jing, Erping Luo, Jing Cai, Shichao Tong, Mingming Zhai, Guanghao Shen, Xin Wang, Zhuojing Luo. " Mechanical Vibration Mitigates the Decrease of Bone Quantity and Bone Quality of Leptin Receptor-Deficient Mice by Promoting Bone Formation and Inhibiting Bone Resorption ", Journal of Bone and Mineral Research, 2016<br>Crossref	12 words — < 1%
35	Taiping Li, Yong Xiao, Zhen Wang, Hong Xiao, Hongyi Liu. "The Mechanism Study of Common Flavonoids on Antiglioma Based on Network Pharmacology and Molecular Docking", Evidence-Based Complementary and Alternative Medicine, 2022<br>Crossref	12 words — < 1%
36	Yanju Zhang, Meiyang Du, Zhouhuiling Li, Xincheng Wang, Mingxin Leng, Yaping Huang, Libin Li, Shi Zhang, Chunjun Li. "The correlation between visceral fat area to skeletal muscle mass ratio and multi-organ insulin resistance in Chinese population with obesity: a cross-sectional study", Research Square Platform LLC, 2023<br>Crossref Posted Content	12 words — < 1%

37 diabetes.diabetesjournals.org
Internet
12 words — < 1%

38 www2.mdpi.com
Internet
12 words — < 1%

39 Jung-In Kim, Min-Jung Kang, Jieun Im, Yeong-Ju Seo, Young-Min Lee, Ji-Hyun Song, Jai-Heon Lee, Mi-Eun Kim. "Effect of king oyster mushroom (Pleurotus eryngii) on insulin resistance and dyslipidemia in db/db mice", Food Science and Biotechnology, 2010
Crossref
11 words — < 1%

40 journals.lww.com
Internet
11 words — < 1%

41 Shasha Tao, Youjing Yang, Jianzhong Li, Hongyan Wang, Yu Ma. "Bixin Attenuates High-Fat Diet-Caused Liver Steatosis and Inflammatory Injury through Nrf2/PPARα Signals", Oxidative Medicine and Cellular Longevity, 2021
Crossref
10 words — < 1%

42 Tianyan Jiang, Haoxiang Guo, Ya-Nan Xia, Yun Liu et al. "Hepatotoxicity of copper sulfide nanoparticles towards hepatocyte spheroids using a novel multi-concave agarose chip method", Nanomedicine, 2021
Crossref
10 words — < 1%

43 bmcmusculoskeletdisord.biomedcentral.com
Internet
10 words — < 1%

44 pubs.rsc.org
Internet
10 words — < 1%

45 Ga-Young Jung, Sae-Bom Won, Juhae Kim, Sookyoung Jeon, Anna Han, Young Hye Kwon.
9 words — < 1%

"Betaine Alleviates Hypertriglycemia and Tau Hyperphosphorylation in db/db Mice", Toxicological Research, 2013
Crossref

---

46 bmcimmunol.biomedcentral.com — 9 words — < 1%
Internet

---

47 Elisa Fabbrini, Shelby Sullivan, Samuel Klein. "Obesity and nonalcoholic fatty liver disease: Biochemical, metabolic, and clinical implications", Hepatology, 2010 — 8 words — < 1%
Crossref

---

48 Jue Liu, Zhifang Deng, Zhijun Yu, Weipin Zhou, Qiong Yuan. "The circRNA circ-Nbea participates in regulating diabetic encephalopathy", Brain Research, 2022 — 8 words — < 1%
Crossref

---

49 Patel, J. H., J. F. L. Cobbold, H. C. Thomas, and S. D. Taylor-Robinson. "Hepatitis C and hepatic steatosis", QJM, 2010. — 8 words — < 1%
Crossref

---

50 Rosen, M.B.. "Gene expression profiling in the lung and liver of PFOA-exposed mouse fetuses", Toxicology, 20070924 — 8 words — < 1%
Crossref

---

51 Shuainan Ma, Di Zhong, Pingwei Ma, Guozhong Li, Wei Hua, Yu Sun, Ning Liu, Linxue Zhang, Weihua Zhang. "Exogenous Hydrogen Sulfide Ameliorates Diabetes-Associated Cognitive Decline by Regulating the Mitochondria-Mediated Apoptotic Pathway and IL-23/IL-17 Expression in db/db Mice", Cellular Physiology and Biochemistry, 2017 — 8 words — < 1%
Crossref

52	Yongnan Li, Shuodong Wu. "Epigallocatechin gallate suppresses hepatic cholesterol synthesis by targeting SREBP-2 through SIRT1/FOXO1 signaling pathway", Molecular and Cellular Biochemistry, 2018<br>Crossref	8 words — < 1%
53	Zili Lei, Huijuan Wu, Yanhong Yang, Qing Hu et al. "Ovariectomy Impaired Hepatic Glucose and Lipid Homeostasis and Altered the Gut Microbiota in Mice With Different Diets", Frontiers in Endocrinology, 2021<br>Crossref	8 words — < 1%
54	aasldpubs.onlinelibrary.wiley.com<br>Internet	8 words — < 1%
55	ijp.mums.ac.ir<br>Internet	8 words — < 1%
56	www.hrjournal.net<br>Internet	8 words — < 1%
57	www.spandidos-publications.com<br>Internet	8 words — < 1%
58	Hwang, J.. "The PPAR@c ligand, rosiglitazone, reduces vascular oxidative stress and NADPH oxidase expression in diabetic mice", Vascular Pharmacology, 200706<br>Crossref	7 words — < 1%
59	Jaborek, Jerad Robert. "Use of Diverse Cattle Breeds to Understand Marbling Development and Growth for the Production of High-Quality Beef.", The Ohio State University, 2020<br>ProQuest	7 words — < 1%

60 Jeong Sook Noh. "Chinese prescription Kangen-karyu prevents dyslipidaemia and oxidative stress in mouse model of type 2 diabetes : Kangen-karyu in type 2 diabetes", Journal of Pharmacy and Pharmacology, 01/2011 — 7 words — < 1%
Crossref

61 Lee, Jung-Ok, Cyril Auger, Dong Hyun Park, Moonkyu Kang, Min-Ho Oak, Kyoung Rak Kim, and Valérie B. Schini-Kerth. "An Ethanolic Extract of Lindera obtusiloba Stems, YJP-14, Improves Endothelial Dysfunction, Metabolic Parameters and Physical Performance in Diabetic db/db Mice", PLoS ONE, 2013. — 7 words — < 1%
Crossref

62 Shi, Dayong, Shuju Guo, Bo Jiang, Chao Guo, Tao Wang, Luyong Zhang, and Jingya Li. "HPN, a Synthetic Analogue of Bromophenol from Red Alga Rhodomela confervoides: Synthesis and Anti-Diabetic Effects in C57BL/KsJ-db/db Mice", Marine Drugs, 2013. — 7 words — < 1%
Crossref

63 pure.rug.nl — 7 words — < 1%
Internet

64 van der Leij, F.R.. "Gene expression profiling in livers of mice after acute inhibition of @b-oxidation", Genomics, 200712 — 7 words — < 1%
Crossref

65 Amanat, Fatima. "In Depth Characterization of Immune Response Against the Spike Protein of SARS-CoV-2 in Response to Infection and mRNA Vaccination", Icahn School of Medicine at Mount Sinai — 6 words — < 1%
ProQuest

66 Hai-Yan Xu, Liang Yu, Ji-Hua Chen, Li-Na Yang, Cui Lin, Xiu-Quan Shi, Hong Qin. "Sesamol Alleviates — 6 words — < 1%

Obesity-Related Hepatic Steatosis via Activating Hepatic PKA Pathway", Nutrients, 2020
Crossref

67	Judit Gil-Zamorano, Roberto Martin, Lidia Daimiel, Kris Richardson et al. "Docosahexaenoic Acid Modulates the Enterocyte Caco-2 Cell Expression of MicroRNAs Involved in Lipid Metabolism1–3", The Journal of Nutrition, 2014<br>Crossref	6 words — < 1%
68	Kokoro Sano, Motoko Kawashima, Akiko Ito, Takaaki Inaba, Kohkichi Morimoto, Mitsuhiro Watanabe, Kazuo Tsubota. "Aerobic Exercise Increases Tear Secretion in Type 2 Diabetic Mice", Investigative Opthalmology & Visual Science, 2014<br>Crossref	6 words — < 1%
69	Shenglan Yang, Chen Chen, Hong Wang, Xiaoquan Rao et al. "Protective Effects of Acyl-coA Thioesterase 1 on Diabetic Heart via PPARα/PGC1α Signaling", PLoS ONE, 2012<br>Crossref	6 words — < 1%

EXCLUDE QUOTES	OFF	EXCLUDE SOURCES	OFF
EXCLUDE BIBLIOGRAPHY	ON	EXCLUDE MATCHES	OFF
